# *Staphylococcus aureus* Survives with a Minimal Peptidoglycan Synthesis Machine but Sacrifices Virulence and Antibiotic Resistance

**DOI:** 10.1371/journal.ppat.1004891

**Published:** 2015-05-07

**Authors:** Patricia Reed, Magda L. Atilano, Renato Alves, Egbert Hoiczyk, Xinwei Sher, Nathalie T. Reichmann, Pedro M. Pereira, Terry Roemer, Sérgio R. Filipe, José B. Pereira-Leal, Petros Ligoxygakis, Mariana G. Pinho

**Affiliations:** 1 Laboratory of Bacterial Cell Biology, Instituto de Tecnologia Química e Biológica António Xavier, Universidade Nova de Lisboa, Oeiras, Portugal; 2 Laboratory of Bacterial Cell Surface and Pathogenesis, Instituto de Tecnologia Química e Biológica António Xavier, Universidade Nova de Lisboa, Oeiras, Portugal; 3 Laboratory of Genes and Development, Department of Biochemistry, University of Oxford, Oxford, United Kingdom; 4 Instituto Gulbenkian de Ciência, Oeiras, Portugal; 5 W. Harry Feinstone Department of Molecular Microbiology and Immunology, Johns Hopkins Bloomberg School of Public Health, Baltimore, Maryland, United States of America; 6 The University of Sheffield, Department of Molecular Biology and Biotechnology, Western Bank, Sheffield, United Kingdom; 7 Merck Research Laboratories IT, Boston, Massachusetts, United States of America; 8 Infectious Disease Research, Merck Research Laboratories, Kenilworth, New Jersey, United States of America; University of Tubingen, GERMANY

## Abstract

Many important cellular processes are performed by molecular machines, composed of multiple proteins that physically interact to execute biological functions. An example is the bacterial peptidoglycan (PG) synthesis machine, responsible for the synthesis of the main component of the cell wall and the target of many contemporary antibiotics. One approach for the identification of essential components of a cellular machine involves the determination of its minimal protein composition. *Staphylococcus aureus* is a Gram-positive pathogen, renowned for its resistance to many commonly used antibiotics and prevalence in hospitals. Its genome encodes a low number of proteins with PG synthesis activity (9 proteins), when compared to other model organisms, and is therefore a good model for the study of a minimal PG synthesis machine. We deleted seven of the nine genes encoding PG synthesis enzymes from the *S*. *aureus* genome without affecting normal growth or cell morphology, generating a strain capable of PG biosynthesis catalyzed only by two penicillin-binding proteins, PBP1 and the bi-functional PBP2. However, multiple PBPs are important in clinically relevant environments, as bacteria with a minimal PG synthesis machinery became highly susceptible to cell wall-targeting antibiotics, host lytic enzymes and displayed impaired virulence in a *Drosophila* infection model which is dependent on the presence of specific peptidoglycan receptor proteins, namely PGRP-SA. The fact that *S*. *aureus* can grow and divide with only two active PG synthesizing enzymes shows that most of these enzymes are redundant *in vitro* and identifies the minimal PG synthesis machinery of *S*. *aureus*. However a complex molecular machine is important in environments other than *in vitro* growth as the expendable PG synthesis enzymes play an important role in the pathogenicity and antibiotic resistance of *S*. *aureus*.

## Author Summary

Peptidoglycan forms the stress-bearing sacculus that prevents lysis of bacteria due to turgor pressure. The integrity of peptidoglycan is therefore essential for bacterial survival and its synthesis is the target of many important antibiotics, such as penicillin. The final steps of peptidoglycan synthesis are catalyzed by penicillin-binding proteins, enzymes that are proposed to work in multi-enzyme complexes. We show that seven of the nine genes encoding peptidoglycan synthesis enzymes can be deleted from the *Staphylococcus aureus* genome without affecting normal growth and cell morphology *in vitro*, identifying the minimal peptidoglycan synthesis machinery of this organism. Identification of minimal machineries is key for synthetic biology efforts towards the design of systems with reduced complexity. However, the non-essential peptidoglycan synthetic proteins are important for survival of *S*. *aureus* in more challenging environments, such as in the presence of antibiotics that target cell wall synthesis or within the host, as shown by the inability of the mutant strain to establish a successful infection and kill *Drosophila* flies.

## Introduction

Many cellular functions are performed by molecular machines that are composed of multiple proteins. Consequently, it is often difficult to determine the precise role of each protein within such a complex. In part this is due to functional redundancy, or to the interdependency of proteins that can result from a recruitment hierarchy or from a requirement of the physical presence of individual proteins to the stability of the entire complex. One approach for the identification of the essential components of a cellular machine consists of determining its minimal protein composition. This information is also key for synthetic biology efforts towards the design of systems with reduced complexity.

One example of a molecular machine that was proposed almost two decades ago [1] but is not yet fully characterized, is the protein complex responsible for the synthesis of peptidoglycan (PG). PG, the main constituent of the bacterial cell wall, is a macromolecule composed of long glycan chains of alternating N-acetylglucosamine and N-acetylmuramic acid units, cross-linked by flexible peptide bridges. The resulting mesh forms a stress-bearing sacculus that envelopes the bacterial cell and prevents lysis due to turgor pressure. The integrity of PG is therefore absolutely essential for bacterial survival and many important antibiotics, such as β-lactams and glycopeptides, target penicillin-binding proteins (PBPs), the enzymes involved in the final stages of PG synthesis. PBPs catalyze the two reactions—transglycosylation and transpeptidation—required to synthesize the glycan strands and to crosslink them via peptides, respectively.

PBPs have been proposed to work in multi-enzyme complexes that may also include cell wall hydrolases and other PG synthesis proteins [1,2]. These complexes would facilitate the coordinated activity of PG synthases and hydrolases, to ensure that growth of the PG mesh occurs without endangering the integrity of the PG sacculus. However, despite years of work from several groups, this hypothetical complex has not been isolated and, particularly in Gram-positive bacteria, we currently lack strong evidence for its existence.

One of the difficulties in studying PG synthesis is the large number of PBPs with apparent redundancy during growth in rich media. For example, the two best-studied bacterial species, *Escherichia coli* and *Bacillus subtilis*, have 12 and 16 PBPs, respectively [3], making it difficult to unravel the specific role of each of these proteins. *Staphylococcus aureus*, a Gram-positive bacterial pathogen, is a particularly good model to study cell wall synthesis, as it contains only four PBPs, PBP1-4 in methicillin-susceptible *S*. *aureus* (MSSA) strains, or five PBPs in methicillin-resistance *S*. *aureus* (MRSA) strains. The latter contain an additional PBP, PBP2A, which is the main determinant for β-lactam resistance due to its low affinity for these antibiotics [4]. The role of the other four, native, staphylococcal PBPs has been reasonably well studied. PBP1 is an essential protein with transpeptidase (TPase) activity, and is involved in cell division and separation [5,6]. PBP2 is the only bi-functional PBP in *S*. *aureus*, with both TPase and transglycosylase (TGase) activities. This protein is essential in MSSA strains, but not in MRSA strains, given that the TPase domain of PBP2 can be replaced by that of PBP2A [7]. However, PBP2 becomes essential in the presence of β-lactams, when cooperation between its TGase domain and the TPase domain of PBP2A is required for survival [8]. PBP3 and PBP4 are non-essential proteins. The function of the former remains largely unknown [9], while the latter was shown to be required for the synthesis of highly cross-linked PG [10]. PBP4 has also been described as important for β-lactam resistance in community-acquired MRSA (CA-MRSA) strains [11].

Besides PBPs, the *S*. *aureus* genome encodes four additional, non-essential, proteins with proven or hypothesized roles in PG synthesis. These are two monofunctional TGases, MGT and SgtA [12–14], and two auxiliary proteins, FmtA and FmtB, which have homology to TPase domains, and therefore are thought to have a role in cell wall synthesis [15–17].

The study of PG synthesis, the target of β-lactam antibiotics, is particularly relevant in *S*. *aureus*, as MRSA strains are currently one of the leading causes of hospital- and community-acquired infections, causing more deaths in the USA than tuberculosis and AIDS combined [18]. The high frequency of antibiotic resistance emphasizes the need for the identification of novel drug targets and of strategies to increase the efficacy of available antibiotics against this pathogen.

Here we describe the construction of a *S*. *aureus* strain encoding the minimal number of PG synthesis enzymes required for survival, which constitute the most relevant targets for the development of antibiotics capable of inhibiting the final stages of PG synthesis. We show that *S*. *aureus* cells are capable of PG synthesis catalyzed solely by the transpeptidase PBP1 and the bi-functional PBP2. However, these cells show loss of resistance to cell wall-targeting antibiotics and decreased virulence in a *Drosophila* infection model, highlighting the important roles of the non-essential enzymes for survival in natural environments, such as a host.

## Results

### *S*. *aureus* encodes nine peptidoglycan synthesis enzymes

**Fig 1:**
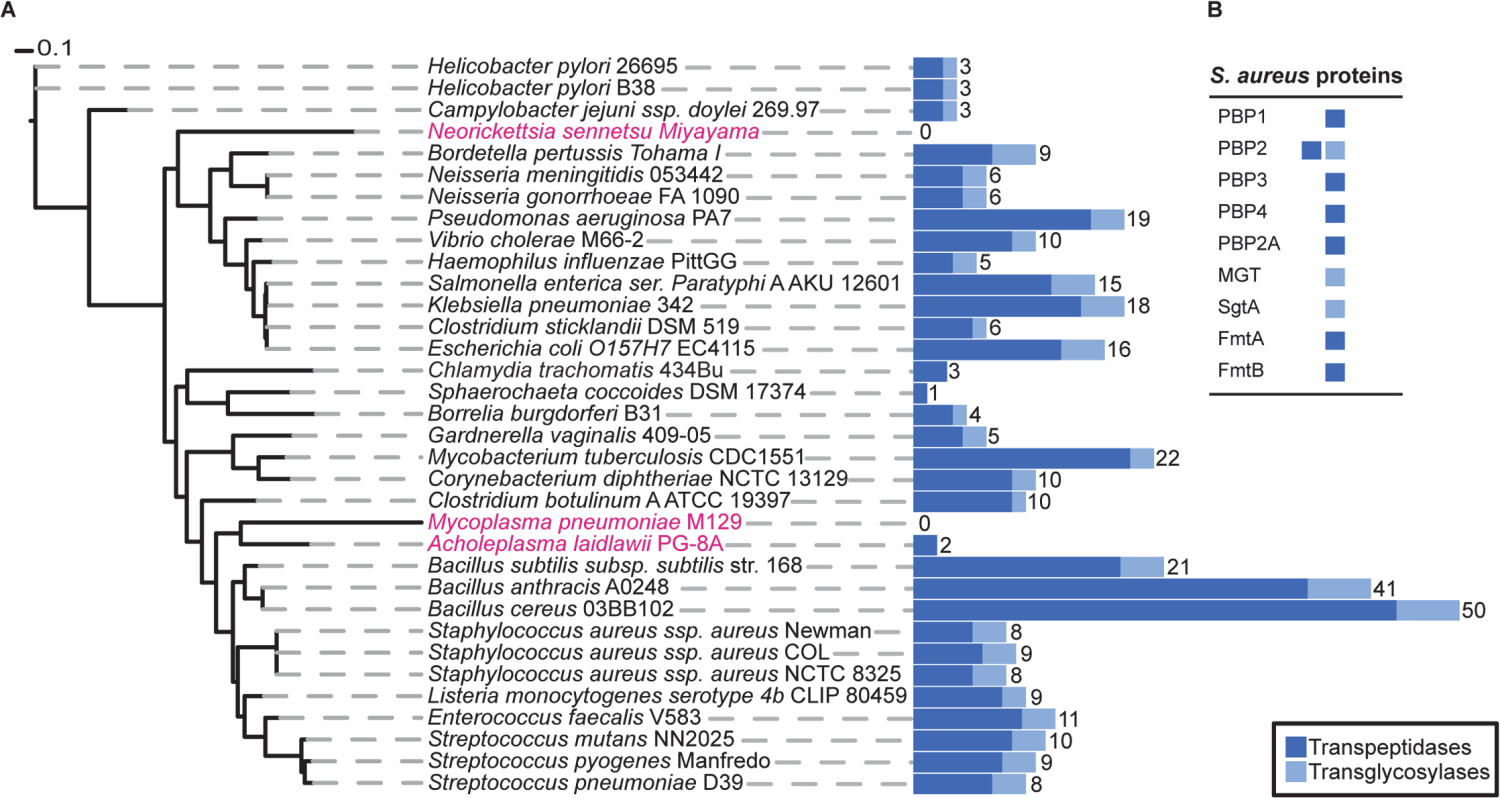
Phylogenetic distribution of peptidoglycan synthesis enzymes across selected bacterial species. **(A)** The blue, stacked bars represent the total number of proteins present in each species that have a transpeptidase (dark blue) or transglycosylase (light blue) domain. The total number of proteins that have at least one of the two domains is displayed numerically. Strains highlighted in pink are bacteria reported not to possess cell wall, although they may produce small but functional amounts of peptidoglycan. The maximum likelihood species tree was calculated using PhyML [53] and concatenated bacterial marker genes identified with the AMPHORA2 [52] software. **(B)** Table showing the peptidoglycan synthesis proteins from *S*. *aureus* and their established or hypothetical activities.

To determine the exact number of proteins likely to play a direct role in PG synthesis in *S*. *aureus*, we used profile Hidden Markov Models (pHMMs) [19], a sensitive bioinformatics approach that can identify both close and distantly related homologues. We scanned the *S*. *aureus* genome with pHMMs based on known transpeptidase and transglycosylase enzymes (SCOP-56601 and SCOP-159832). This analysis identified all nine previously known enzymes (one bi-functional) with a demonstrated or suggested role in PG synthesis (see Introduction) comprising a total of ten active domains, 7 transpeptidases (TPases) and 3 transglycosylases (TGases) (Fig 1).

Next, we extended this analysis to 1295 organisms from the bacterial kingdom to determine the absolute minimal number of PG synthesis domains present in a bacterium whose genome has been fully sequenced. Fig 1 shows the results for selected species (mainly disease causing and model organisms) and illustrates the variety in the number of enzymes predicted to be involved in PG synthesis. The number of encoded TPases and TGases varied dramatically from as many as 62, encoded by *Streptosporangium roseum*, to none in intracellular pathogens such as *Mycoplasma pneumoniae*, which are often considered to be lacking PG. The lowest number of enzymes in free-living bacteria was found in the non-pathogenic bacterium, *Sphaerochaeta coccoides* (1 TPase), though studies of its PG composition have not been described. Among species with known PG composition, *Helicobacter pylori* has the smallest set of PG synthesis enzymes, with one bi-functional enzyme (PBP1) and two monofunctional TPases (PBP2 and PBP3) [20]. In this organism PBP2 is essential for viability [21] and depletion of PBP1 or PBP3 causes significant morphological defects [22].

### A *S*. *aureus* strain with a minimal PG synthesis machinery grows normally and displays normal cell morphology

The non-essential nature of PBP3, PBP4 and PBP2A for survival of *S*. *aureus* has been previously described [4,9,10,23]. MGT and SgtA as well as FmtA and FmtB are also individually dispensable, which could be due to redundant functions of these proteins [12,15,17]. In order to assess the effect of the simultaneous lack of these enzymes upon growth and survival, we sequentially deleted from the chromosome of the MRSA strain COL, the genes *pbp3*, *pbpd* (encoding PBP4), *mgt*, *sgtA*, *fmtA*, *fmtB*, and *mecA*. In-frame, markerless deletions of each gene were successively constructed, as outlined in S1 Fig, to produce the strain COL MIN. This strain contains a minimal PG synthesis machinery, as it encodes only two of the nine known proteins with PG synthesis activity—PBP1 and PBP2. Western blotting of whole cell extracts was performed to confirm the absence of PBPs (S2 Fig) and all gene deletions and lack of gene duplications were confirmed by PCR (S2 Fig) and genome sequencing (see below).

**Fig 2:**
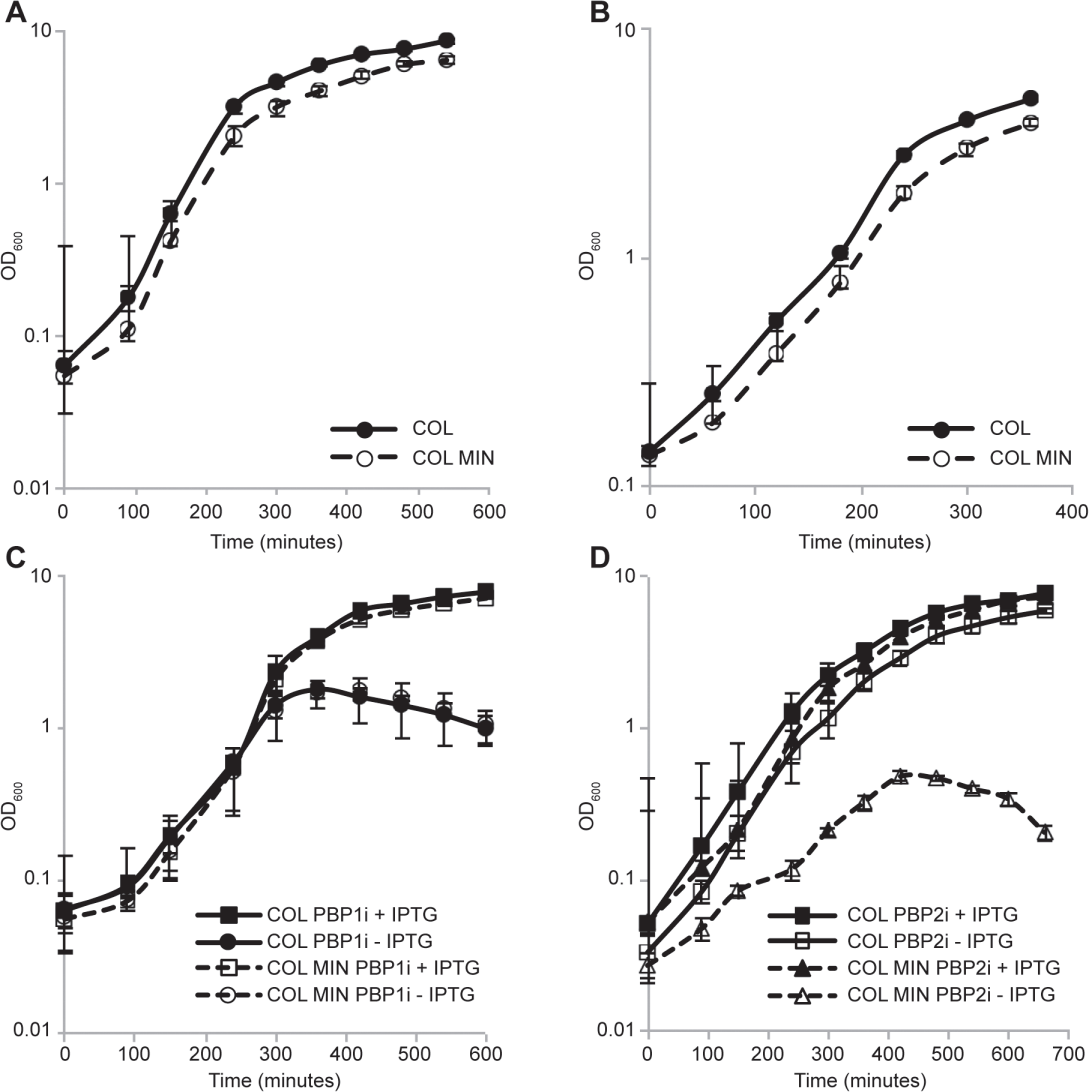
*S*. *aureus* minimal mutant strain COL MIN displays normal growth and requires PBP1 and PBP2 for survival. **(A)** Growth of the parental strain COL and the minimal mutant strain COL MIN was followed in rich liquid medium by monitoring the absorbance at OD_600nm_. The mutant strain COL MIN (doubling time 40 min) showed similar growth to the parental strain COL (doubling time 36 min). **(B)** Growth of COL and COL MIN was followed in minimal medium by monitoring the absorbance at OD_600nm_. The mutant strain COL MIN (doubling time 67 min) showed similar growth to the parental strain COL (doubling time 61 min). **(C)** Depletion of PBP1 from COL PBP1i and COL MIN PBP1i, in which PBP1 expression is under the control of the IPTG inducible P_*spac*_ promoter, by growing cells in the absence of IPTG, led to a halt in cell growth and subsequent drop in optical density indicating PBP1 is essential for survival of both the parental and mutant strains. **(D)** In the absence of PBP2, strain COL PBP2i (parental strain COL with PBP2 expression under the control of the IPTG inducible P_*spac*_ promoter) continues to grow. However, depletion of PBP2 from COL MIN PBP2i causes arrest in growth indicating PBP2 is essential for growth of COL MIN. Averages of three independent replicates are shown and error bars show standard deviations.

Surprisingly, *S*. *aureus* was able to grow normally both in rich medium as well as in minimal medium in the absence of seven PG synthesis enzymes (Fig 2A and 2B), requiring only PBP1 and PBP2 for viability under laboratory conditions.

PBP1 is an essential protein [6], thus in order to verify if this was also the case in COL MIN, we constructed strain COL MIN PBP1i, in which the native copy of the gene encoding for PBP1 was placed under the control of the IPTG-inducible P_*spac*_ promoter in the COL MIN background. Growth analysis showed that upon depletion of the protein (via growth in the absence of IPTG), cells do not grow, confirming the essentiality of PBP1 in COL MIN (Fig 2C). Given that PBP1 is part of a large operon, polar effects were ruled out by introducing a replicative plasmid expressing PBP1 into COL MIN PBP1i and showing that the resulting strain (COL MIN PBP1i pSKP1) is able to grow in the absence of IPTG (S3 Fig).

PBP2 expression in the absence of either PBP2A [7] or MGT [12] has been previously described as essential. Thus we placed PBP2 expression under the control of the IPTG-inducible P_*spac*_ promoter in the background of COL MIN and confirmed that the resulting strain does not grow in the absence of the inducer (Fig 2D). Although the gene encoding PBP2 is part of an operon, the other gene in the operon, *recU*, is not essential [24].

### *S*. *aureus* COL MIN adapts to loss of peptidoglycan synthesis enzymes by activating the cell wall stress stimulon and downregulating autolysins

To determine if COL MIN had suppressor mutations that allowed survival in the absence of seven PG synthesis enzymes, we sequenced its entire genome (Table A in S1 Text). This approach confirmed the deletion of *pbp3*, *pbpd*, *mgt*, *sgtA*, *fmtA*, *fmtB*, and *mecA* in COL MIN. A total of fifteen mutations were identified in COL MIN that were absent in the parental strain COL. Of these, 9 were very close to the excision sites corresponding to deletions of *sgtA*, *mgt*, *fmtB* and *mecA* and are therefore unlikely to have any effect upon other genes. Five genetic differences between COL MIN and the parental strain COL were SNPs in non-coding regions of the genome, close to genes of unknown function or unrelated to peptidoglycan synthesis. Only one SNP was in a coding region, in the gene encoding the molybdenum-binding protein ModA. In Gram-negative organisms this protein is involved in the uptake of nutrients including metals such as molybdenum [25] and therefore it is unlikely that the ModA SNP found in COL MIN promotes new PG synthesis activities or decreases PG autolysis.

Given that genome sequencing did not elucidate any mechanism of adaptation of COL to the loss of seven proteins required for peptidoglycan synthesis, we compared the over all gene expression profile of COL and COL MIN by total RNA sequencing (RNA-Seq). Expression of approximately 155 genes was significantly altered (p<0.05 and over two-fold difference, Table B in S1 Text). Of these, 59 are part of the large lysogenic prophage L54a that may be excised in a subpopulation of COL MIN cells. Analysis of the remainder showed upregulation of the *vraSRF* operon, which controls the so-called “cell wall stress stimulon”, known to be regulated in response to cell wall damage [26]. The *vraS* and *vraR* genes were up regulated more than 3-fold in COL MIN compared to COL. Almost half of the 46 genes shown to be regulated by the VraSR system [26] also showed increased expression of the transcripts and of these 11 showed a fold-difference in expression of greater than 2-fold. Another relevant observation was the down regulation of a number of known or putative PG hydrolases (*sle1* (2.3 fold), *lytD* (1.7 fold), SACOL2298 (1.3 fold), *sceD* (3.6 fold), *isaA* (2.8 fold) and *lytM* (3.3 fold)), presumably to reestablish a required balance between PG synthesis and autolysis. Interestingly, none of the proteins involved in the synthesis of PG precursors (Mur A-G, FemABX, MraY) showed differences in gene expression in the mutant strain.

### *S*. *aureus* COL MIN is capable of synthesizing PG that sustains normal cell morphology

**Fig 3:**
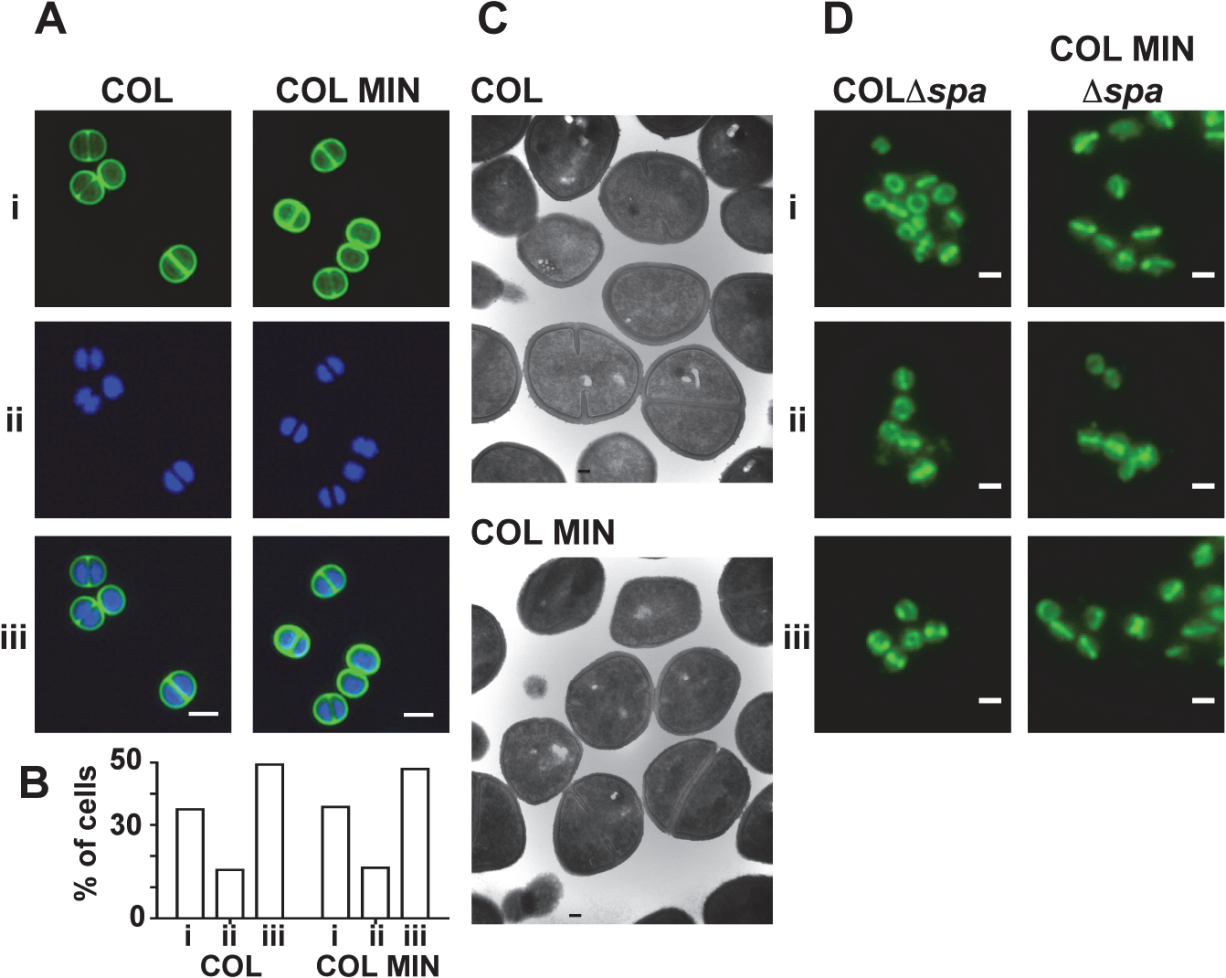
*S*. *aureus* strain COL MIN displays normal morphology and correct localization of PBP1 and PBP2. **(A)** Structured illumination microscopy images of cells incubated with (i) Van-FL, and (ii) Hoechst 33342 to label the cell wall and DNA, respectively, show no difference between the parental strain COL and COL MIN, (iii) overlay of Van-FL and Hoechst labeled cells. Scale bars represent 1μm. (**B**) Percentage of cells with complete septa (i), partial septa (ii) and no septa (iii) in COL (n = 333) and COL MIN (n = 223) strains. **(C)** Representative electron microscopy images of COL and COL MIN show that cells retain a normal shape and septum placement in the absence of seven PG synthesis enzymes. **(D)** Localization of PBP1 (i), PBP2 (ii) and FtsZ (iii), by immunofluorescence, in COLΔ*spa* and COL MIN Δ*spa* cells shows that the three proteins localize to the septum in the COL MIN strain, similarly to the parental strain COL. FtsZ was used as a control for septal localization. Strains lacking the *spa* gene were used for immunofluorescence experiments as the *spa* gene product Protein A binds with high affinity to IgG molecules. Scale bar represents 1μm.

To determine if the lack of seven PG synthesizing enzymes in COL MIN resulted in cell morphology defects, cells were examined with transmission electron microscopy and with super-resolution fluorescence microscopy using the DNA dye Hoechst 33342 and a fluorescent derivative of vancomycin (Van-FL), which labels PG by binding the terminal D-Ala-D-Ala residues of the muropeptides. COL MIN showed no morphological changes compared to the parental strain COL, when observed by Structured Illumination Microscopy (SIM) and electron microscopy (Fig 3A and 3C). The fraction of cells with septa (COL 38.1%, COL MIN 37.6%) and the average cell diameter (COL 1.06 μm ± 0.08, COL MIN 1.07 μm ± 0.08) remained unchanged. These results suggest that, in COL MIN, the remaining PG synthesis enzymes were sufficient to maintain the normal morphology of the cell. In wild type *S*. *aureus*, both PBP1 and PBP2 have been shown to localize to the division septum [5,27], where cell wall synthesis takes place [28,29]. Given that PBP1 or PBP2 have been reported to interact with other PG synthesis enzymes in bacterial two hybrid assays [12,30], it was possible that these proteins required the presence of other members of a putative cell wall synthesis complex for correct localization. We therefore investigated the localization of PBP1 and PBP2 in COL MIN by immunofluorescence, in strains lacking the *spa* gene given that its gene product, Protein A, binds with high affinity to IgG molecules, and using FtsZ as a control for septal localization. Both PBP1 and PBP2 maintained their normal septal localization in the absence of the other PG synthesis enzymes (Fig 3D) indicating that they do not depend upon them for correct localization.

We also verified whether the presence of PBP1 and PBP2 was sufficient for synthesis of PG with a composition similar to the parental strain COL. For that purpose, PG was purified from the parental strain COL and COL MIN and digested with muramidase to isolate muropeptides. When muropeptides were analyzed by reverse-phase-high pressure liquid chromatography (HPLC), the major difference between the two strains was a reduction in highly cross-linked muropeptides, (peak V and above, S4 Fig). This phenotype was expected as PBP4, and to a smaller extent FmtA, both absent in COL MIN, were previously shown to be responsible for the synthesis of highly cross-linked PG in *S*. *aureus* [17,31]. Analysis of single deletion mutants in genes encoding PG synthesis enzymes confirmed that the decrease in crosslinking in COL MIN was due to the lack of PBP4 and FmtA (S4 Fig).

To analyze the glycan strands from COL and COL MIN, we performed a sequential digestion of the PG with lysostaphin (to cleave the pentaglycine bridge between different glycan strands) followed by LytA amidase (to remove the stem peptides from the glycans) as previously described [32]. The glycans were then analyzed by HPLC and showed only minor differences in length or composition (S5 Fig). We have previously reported that PBP2 is the major transglycosylase in *S*. *aureus* [12] and the results here confirm that removal of MGT and SgtA, the other enzymes with transglycosylase activity, has no major effect upon glycan chain length or composition. Thus our results show that *S*. *aureus* can form a functional cell wall using only PBP1 and PBP2.

Importantly, the minimal strain described above was constructed in the background of MRSA strain COL. MRSA strains are adapted to live in the presence of β-lactam antibiotics, i.e., in conditions where their native transpeptidases are inactivated. We therefore questioned if the genes *pbp3*, *pbpd*, *mgt*, *sgtA*, *fmtA* and *fmtB* could also be deleted in an MSSA strain, which is susceptible to β -lactams. S6 Fig shows that strain Newman MIN, which encodes only PBP1 and PBP2 as PG synthesis enzymes, was also viable in rich and minimal medium.

### *S*. *aureus* containing a minimal set of PG synthesizing enzymes is exquisitely sensitive to cell wall targeting antibiotics

We have described above that deletion of genes encoding seven of the nine identified PG synthesizing enzymes from the genome of *S*. *aureus* has little effect upon viability, growth, morphology or PG composition in rich medium. However, *in vitro* growth assays are not sufficient to study the fitness of a pathogen such as *S*. *aureus*, best known for its ability to resist various antibiotics, in particular β-lactams, which target cell wall synthesis. Therefore we measured the minimum inhibitory concentration (MIC) of an array of antibiotics for COL and COL MIN, Newman and Newman MIN. In the absence of the non-essential PG synthesis proteins, *S*. *aureus* became exquisitely sensitive to antibiotics directly targeting PG synthesis enzymes (β-lactams and moenomycins), but retained normal resistance to antibiotics targeting other pathways (Table 1). For example, in COL, oxacillin resistance is only slightly reduced (2 fold) in the combined PBP3, PBP4, MGT and SgtA mutant (Table C in S1 Text). Upon additional deletion of *fmtA*, resistance to antibiotics that target TPases decreases dramatically, even though the strain retains PBP2A and PBP2, the two enzymes capable of sustaining PG synthesis in the presence of β-lactams [7,8]. FmtA has been previously described to have a role in β-lactam resistance [17], as interruption of the corresponding gene resulted in an 8-fold decrease in oxacillin resistance. Here, deletion of *fmtA* in a background already lacking PBP3, PBP4, MGT and SgtA led to a greater than 100-fold decrease in the oxacillin MIC, indicating that there is a cumulative effect on resistance upon deletion of the genes encoding these proteins (Table C in S1 Text).

**Table 1:** MICs of antibiotics for parental and mutant strains.

Antibiotic	COL MIC (μg/ml)	COL MIN MIC (μg/ml)	Newman MIC (μg/ml)	Newman MIN MIC (μg/ml)
Oxacillin (TPases)*	800	3.125	4	0.0078
Imipenem (PBP1)	50	0.39	10	10
Cefotaxime (PBP2)	500	3.9	4	0.0625
Cephradine (PBP3)	500	3.9	4	0.5
Cefoxitin (PBP4)	500	3.9	0.5	0.5
Flavomycin (TGases)	2.5	0.31	2.5	0.325
Bacitricin	50	50	50	50
Vancomycin	2.5	2.5	2.5	2.5
Lysostaphin	0.125	0.125	0.125	0.125
Fosfomycin	1000	1000	1000	1000
Chloramphenicol	10	10	10	10
Nalidixic Acid	100	100	100	100

* preferential target of antibiotic shown in parenthesis

### COL MIN is more susceptible to lysozyme and is attenuated for virulence in a *Drosophila* infection model

Finally, we tested if COL MIN was able to establish a successful infection, using *Drosophila melanogaster* as a model organism. *D*. *melanogaster* has been used to show that the composition of the cell surface of *S*. *aureus* has a crucial role in the ability of these bacteria to avoid host recognition and survive inside the host [33].

**Fig 4:**
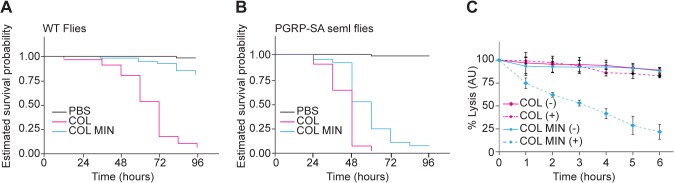
*S*. *aureus* COL MIN showed attenuated virulence in a *Drosophila* infection model and increased susceptibility to lysozyme. **(A, B)** Estimated survival curves for wild type (WT) and PGRP-SA mutant (*seml*) flies infected with COL and COL MIN *S*. *aureus* strains or PBS (to monitor the physical effects of the injection *per se*). WT flies strongly succumbed to infection with COL by 96 hours whereas 88% of WT flies infected with COL MIN survived. Curves were statistically separable, log-rank test P<0.05. At least 90% of the PGRP-SA-deficient flies were killed by WT bacteria (within 60 hours) and by COL MIN mutant strain (within 96 hours). Curves were statistically separable, log-rank test P<0.05. (**C)** Bacterial cell lysis monitored through the decrease of OD_600_ was determined for COL and COL MIN strains in the presence (+) or absence (-) of lysozyme (300 μg/ml). The minimal strain showed increased cell lysis in the presence of lysozyme. Data shows mean with 95% confidence intervals of three independent biological repeats.

To determine whether the deletion mutant strain was affected in virulence, we injected *Drosophila* flies with equal numbers of COL and COL MIN *S*. *aureus* cells and determined the ability of both strains to kill *Drosophila*. Over 90% (n≈90) of flies injected with the parental strain COL were killed within 96 hours, while only 12% of the flies infected with COL MIN were killed during that period (Fig 4A). To test whether decreased virulence was due to impaired ability of COL MIN to avoid host recognition, we used a *Drosophila* strain mutant for the peptidoglycan receptor *pgrp-sa*. A functional PGRP-SA is paramount for host survival against *S*. *aureus* infection, binding to *S*. *aureus* peptidoglycan and leading to Toll pathway activation, with the consequent production of antimicrobial peptides [34,35]. Injection of COL MIN in *Drosophila seml* mutants (which express a non-functional PGRP-SA) resulted in killing of approximately 95% of the flies (Fig 4B), showing that the impaired ability of COL MIN to kill *Drosophila* wild type flies was likely due to decreased ability to avoid recognition by PGRP-SA. Accordingly, COL MIN was unable to propagate in wild type *Drosophila* flies, but it was able to propagate as well as COL in *Drosophila seml* mutants (S7 Fig). Furthermore, the inability of COL MIN to propagate in wild type *Drosophila* flies was not due to higher production of antimicrobial peptides, as expression of drosomycin was significantly lower when flies were infected with COL MIN than when flies were infected with COL (S7 Fig).

Even in the absence of PGRP-SA (i.e. in *Drosophila seml* mutants), the killing curves of COL and COL MIN were statistically separable (Fig 4B). This could be due to the slightly reduced growth rate of COL MIN or could suggest the existence of a second factor, besides enhanced recognition by PGRP-SA, which contributes to the reduced virulence of COL MIN. We therefore determined the susceptibility of COL MIN and COL to lysozyme, a PG hydrolase, and found sensitivity to be increased in COL MIN (Fig 4C), possibly due to the decreased degree of PG crosslinking [36,37], suggesting that the reduced virulence of COL MIN is due to a decreased ability to avoid host recognition and to resist host defense mechanisms such as the action of bacteriolytic enzymes.

## Discussion

One way to determine the minimal required composition of complex multi-protein machines is through genetic screens, which have been used in various bacterial species, including *S*. *aureus* [38], to identify all essential genes. However, those screens identify genes that are dispensable when disrupted one at a time, but not genes with redundant functions that cannot be simultaneously deleted from the genome due to synthetic lethality. To identify such genes, deletion of multiple genes in various combinations is required, a process that can be used both to identify the key proteins required for specific pathways, and for efforts to build a minimal cell that contains the smallest set of essential genes.

In this work we have identified the minimal components of the molecular machinery required for PG synthesis in *S*. *aureus*. In the late 1990´s, J. Höltje proposed a three-for-one model for synthesis of Gram-negative PG, which stated that it required the concerted action of multiple enzymes that simultaneously polymerize and hydrolyze the PG, to allow insertion of new material without compromising the integrity of the stress-bearing PG sacculus that surrounds the bacterial cell [1,2]. According to the model, this concerted action would be facilitated through the formation of multi-enzyme complexes, which would allow better coordination of the proteins involved in PG synthesis [1]. The work that led to this model was performed in *Escherichia coli*, a Gram-negative bacteria, which has a very thin layer of PG [39], and since then genetic, biochemical and cell biological approaches have shown that various PG synthesis enzymes from *E*. *coli* are indeed able to interact with each other (reviewed in [40]). Despite this progress we are still far from understanding the PG synthesis complex in a level of detail similar to what has been achieved for other enzyme complexes, for example those involved in the synthesis of DNA or RNA [41,42]. Even less information has been obtained for proteins synthesizing PG in Gram-positive bacteria that contain a thick layer of PG [43]. Interactions between PBPs of Gram-positive organisms have been suggested, based on bacterial two-hybrid assays [12,30], but so far, to the best of our knowledge, no interactions between Gram-positive PG synthesis enzymes have been confirmed biochemically.

In this work, we have generated a mutant *S*. *aureus* strain (COL MIN) by deleting seven of the nine known genes encoding proteins with PG synthesis activity. Remarkably, the resulting cells are not only viable, but grow almost as well as the parental strain in rich as well as minimal medium, and have normal morphology and cell size, as seen by super resolution and electron microscopy. In this mutant, the PG is less extensively cross-linked, as would be expected for a strain lacking PBP4 [31], but otherwise, its composition is similar to the wild type strain. One could expect that if a large complex requiring some or all of these enzymes was required for PG synthesis, its activity would be impaired in the COL MIN strain, which is not what was observed. Furthermore, it is often the case that different components of multi-enzyme complexes depend upon each other for correct localization, as has been shown for the cell division machinery, the divisome. This multi-protein complex is built by recruiting proteins to the division site in a specific order, with proteins crucially depending on the presence of earlier ones for correct localization [44,45]. In COL MIN, the two remaining known PG synthesis enzymes, PBP1 and PBP2, are correctly localized at the septum and therefore do not depend on other PG synthesis enzymes for correct localization.

Currently, we cannot rule out the possibility that other, as yet unidentified, proteins are involved in PG synthesis of *S*. *aureus* but show no homology to known TPases or TGases and were therefore missed in our bioinformatics analysis. In *B*. *subtilis*, which encodes no canonical monofunctional glycosyltransferases, it is possible to remove all class A PBPs, and thus all known TGase activities [46]. This could suggest that other proteins capable of synthesizing PG but lacking homology to known TGases are yet to be identified. A similar observation has been made in *E*. *faecium*, which can also survive in the absence of its three Class A PBPs, suggesting that polymerization of the PG glycan strands in this mutant is catalyzed by an unknown transglycosylase enzyme [47].

Whole genome sequencing of COL and COL MIN identified minor differences between the two strains, but no gene duplications or chromosomal rearrangements. Fifteen SNPs were identified, in the scar regions corresponding to the deleted genes, in non-coding regions and in the gene encoding the molybdenum-binding protein ModA, which is part of an ABC transporter system for the uptake of nutrients [25] and is not involved in peptidoglycan synthesis. None of the mutations were present in genes likely to act as suppressors for the lack of the seven deleted genes encoding proteins involved in PG synthesis. However, transcriptome-wide analysis of the mutant COL MIN compared to the parental strain COL, by total RNA sequencing, revealed a down-regulation of a number of PG hydrolases in response to removal of seven of the nine PG synthetic enzymes. It was previously shown that down-regulation of PBP2 expression caused a concurrent reduction in transcript level of the major autolysins Atl and Sle1 [48], and it is thought that transcriptional regulation between cell wall synthetic and hydrolytic enzymes exists. Therefore it is not surprising that we note a down-regulation of some of the other PG hydrolases of *S*. *aureus* in the absence of seven PG synthetic enzymes. RNA-Seq data also confirmed that transcription levels of PBP1 and PBP2 remain unchanged in the mutant strain COL MIN indicating that normal levels of these proteins are sufficient in order to perform normal CW synthesis in the absence of the other enzymes. None of the genes involved in synthesis of the PG precursor Lipid-II had altered transcript levels, strengthening the suggestion that PBP1 and PBP2 can function normally to build PG in the strain COL MIN.

In summary, this study has identified the minimal machinery required for PG synthesis in *S*. *aureus*. While our goal was not to disprove the existence of a multi-enzyme PG synthesis complex, our results demonstrate the plasticity of this process. Apparently, *S*. *aureus* can synthesize its PG by using just two synthesis protein, PBP1 and PBP2, which performs both transpeptidation and transglycosylation reactions. In agreement with our data, a recent report described that in *Caulobacter crescentus*, a Gram-negative organism, only one of the five bi-functional PBPs encoded by this organism is required for growth and normal morphogenesis [49]. This study however did not investigate the requirement of the *C*. *crescentus* monofunctional transglycosylase MtgA.

The existence of simple PG synthesis machineries is further supported by our comprehensive search for PG synthesizing proteins in sequenced bacterial genomes. Among the 1295 species analyzed, we found one free-living bacteria with even fewer proteins than we have in COL MIN, the Gram-negative termite hindgut bacterium *Sphaerochaeta coccoides*, which encodes for only one TPase. However, to the best of our knowledge, studies of the cell wall of this organism have not been performed. Among bacteria with characterized PG, the Gram-negative pathogenic bacterium *Helicobacter pylori* has the minimal set of PG synthesis enzymes, with two monofunctional TPases and one bi-functional enzyme [20]. However, the vast majority of species have a higher number of PG synthesis proteins, suggesting that optimal growth in challenging environments requires a more complex set of these proteins. Accordingly, although *S*. *aureus* can apparently survive relying solely upon the activity of PBP1 and PBP2 for PG synthesis, we have shown that non-essential PG synthesis enzymes are required for survival in more complex habitats, i.e., in a host infection model, or in the presence of antibiotics. In fact, COL MIN is exquisitely sensitive to antibiotics that target enzymes with TPase or TGase activities and it is also severely affected in its ability to establish a successful infection and kill *Drosophila* flies. Further support for the role of PBPs in virulence comes from a recent study showing that inhibition of PBPs by nafcillin reduces virulence of MRSA in a murine subcutaneous infection model [50]. The specific role of each of the cell wall synthesis enzymes missing in COL MIN for survival within the host is not known. However, we have shown that the inability of COL MIN to successfully kill *Drosophila* flies is essentially reversed if the host lacks the peptidoglycan recognition protein PGRP-SA, which indicates that impaired virulence is most likely due to alterations in the cell surface that affect host recognition of the peptidoglycan. Some contribution to impaired virulence may come from increased susceptibility to the bacteriolytic effects of lysozyme, a PG hydrolase expressed by many host organisms.

By removing seven of the nine PG synthesis enzymes present in *S*. *aureus* cells, we have highlighted the redundancy in this process in rich and minimal medium, but not in more challenging environments such as in the presence of cell-wall targeting antibiotics or within the host.

## Materials and Methods

### Bacterial strains and growth conditions

The bacterial strains used in this study are listed in Table D in S1 Text and details of their construction are outlined in the supplementary materials and methods (S1 Text). Plasmids and primers used in this study are listed in Tables E and F in S1 Text, respectively. *S*. *aureus* strains were grown at 37°C in Tryptic soy broth medium (TSB; Difco) or on Tryptic soy agar (TSA; Difco) supplemented with appropriate antibiotics when required (erythromycin 10 μg/ml, chloramphenicol 10 μg/ml or tetracycline 5 μg/ml; Sigma-Aldrich) or with 0.5 mM isopropyl-β-D-thiogalactopyranoside (IPTG; VWR). For growth studies in minimal media cells were grown in SSM9PR minimal media containing 1 x M9 salts, 2 mM MgSO_4_, 0.1 mM CaCl_2_, 1% glucose, 1% casaminoacids, 1 mM Thiamine-HCl and 0.05 mM nicotinamide at 37°C. *E*. *coli* strains were grown at 30°C or 37°C in Luria-Bertani broth medium (LB broth; Difco), on LB agar (Difco), supplemented with 100 μg/ml ampicillin (Sigma-Aldrich) or 50 μg/ml kanamycin (Sigma-Aldrich), 40 μg/ml 5-bromo-4-chloro-3-indolyl-β-D-galactopyranoside (X-gal; VWR) and 0.5 mM IPTG when required.

### Bioinformatics analysis

Analysis of the distribution of PBPs and enzymes containing PBP-like domains was performed using domains SCOP-56601 and SCOP-159832 in the Superfamily database version 1.75 [51], as proxies for transpeptidases and transglycosylases respectively. A maximum likelihood tree was constructed using the software AMPHORA2 [52] and PhyML [53] following the procedure described by Wu *et al* [54].

### Genome sequencing

Genomic DNA was extracted from individual cultures of the parental, intermediate and minimal strains, and sequenced using the Illumina HiSeq system at Beijing Genomics Institute or the Illumina MiSeq system at Instituto Gulbenkian de Ciência, Oeiras, Portugal. 300-bp paired end reads with over 100x average coverage were generated. Sequence reads were assembled with SeqMan NGen 11 software using the COL genome (NCBI Accession NC_002951.2) as a reference. The variations that were detected in the parental COL strain were filtered to identify the mutations occurring in the intermediate and minimal strains. Low quality variations with read frequencies below 50% were removed from the dataset.

### Total RNA extraction, high-throughput RNA sequencing and data analysis

Overnight cultures from isolated colonies were diluted 1/200 into fresh medium and incubated at 37°C with aeration to exponential phase (OD_600_ ≈ 0.6). Cells were harvested, and RNA was extracted using the Qiagen RNA Easy Kit. Integrity of RNA samples was evaluated using a 2100 Bioanalyzer (Agilent Technologies). Total RNA samples (40 μg) were sent to GATC Biotech AG, Germany for library preparation (including rRNA depletion and DNase treatment) and sequencing of libraries was performed using an Illumina HiSeq platform (paired end, 2 x 125bp read length). The RNA-Seq reads were aligned to the COL reference genome (NC_002951) using Bowtie [55], generating genome/transcriptome alignments. Cufflinks was used to process the raw data, identifying and quantifying the transcripts from the preprocessed RNA-Seq alignment-assembly. Cuffdiff was used to compare the transcripts from COL and COL MIN to determine the differential expression levels between the two samples.

### Growth analysis of *S*. *aureus* strains

Growth of parental and mutant strains in liquid culture was analyzed by diluting overnight cultures 1/200 into fresh medium (TSB). Cultures were incubated at 37°C with shaking and OD_600_ was monitored. Growth of strains with *pbp1* or *pbp2* under the control of the IPTG-inducible promoter P_*spac*_ was monitored by first incubating strains overnight at 37°C in TSB medium supplemented with appropriate antibiotics and 0.5 mM IPTG. Cells were harvested the following day, washed three times with fresh TSB lacking IPTG and used to inoculate media with and without IPTG. Cultures were incubated at 37°C with agitation and the OD_600_ was recorded.

### Determination of antibiotic susceptibility

Determination of the MIC to an array of antibiotics was performed in TSB by micro-dilution in 96-well plates. Overnight cultures of parental and mutant strains were added at a final cell density of 5x10^3^ CFU/ml to wells containing 2-fold dilutions of each antibiotic. Plates were incubated at 37°C for 24 or 48 hours and the MIC was recorded as the lowest concentration of antibiotic that inhibited bacterial growth. Results are the average of six independent MIC determination experiments.

### Peptidoglycan purification and analysis

Peptidoglycan was prepared from exponentially growing cells as previously described [56]. Muropeptides were prepared by digestion with mutanolysin and glycan strands were isolated from purified peptidoglycan by sequential digestion with recombinant lysostaphin (1 μg/ml, Sigma) and purified pneumococcal amidase (LytA, 50–100 μg/ml) essentially as previously described [32] and detailed in the supplementary materials and methods (S1 Text).

### Microscopy

Parental and mutant strains were labeled with DNA dye Hoechst 33342 (1 μg/ml, Invitrogen) and the cell wall dye Van-FL (Invitrogen) and visualized by fluorescence Structured Illumination Microscopy (SIM). Immunolabelling of COLΔ*spa* and COL MINΔ*spa* cells with antibodies specific for PBP1, PBP2 and FtsZ was performed essentially as previously described [5]. Electron microscopy analysis of parental and mutant strains was performed as previously described [57]. All microscopy procedures are detailed in the Supplementary materials and methods (S1 Text).

### Determination of lysozyme susceptibility

*S*. *aureus* cells (overnight culture) were harvested by centrifugation, washed once with phosphate saline solution (PBS, 10 mM Na_2_PO_4_/ 150 mM NaCl, pH 6.5), and adjusted to an OD_600_ of 0.4 in 50 ml of PBS. Cell suspensions were split in two and incubated with or without lysozyme (final concentration 300 μg/ml; Sigma) for 6 h with shaking at 30°C. Bacterial lysis was monitored by following the OD_600_ and the percentage of bacterial lysis was calculated by using the following formula: (OD_T_/OD_T0_) × 100, where OD_T0_ indicates OD of the culture for time zero and OD_T_ is the OD of the culture after incubation with lysozyme at a certain time point.

### Infection of *Drosophila* flies with *S*. *aureus* cells

Isogenized 25174 *Drosophila* flies (Bloomington Drosophila Stock Center) were used as wild-type background and PGRP-SA^*seml*^ flies [58,59] were used as a loss of function strain for the *Drosophila* PGRP-SA receptor. All flies were kept on maize malt molasses food in bottles and reared at 25°C before infection.

To infect flies, *S*. *aureus* COL and COL MIN strains were cultured in TSB for 16 hours; cells were harvested by centrifugation (4000 rpm for 7 minutes) and washed in sterile phosphate buffered saline (PBS). Washed bacterial cells were again centrifuged and re-suspended in PBS to an optical density of approximately 0.330 (Thermo Scientific NanoDrop 1000 spectrophotometer). The inoculants containing *S*. *aureus* COL and COL MIN strains were further diluted 450-fold in PBS. Thirty CO_2_-anaesthetized female *25174* or PGRP-SA*^seml^* flies (aged 2–4 days) were infected with 13.2 nl of bacterial cells suspension (or with PBS control), directly injected into the haemolymph through the dorsolateral region of the thorax, using a micro-injector (Drummond Scientific Nanoinject II). The number of viable bacteria cells injected per fly was approximately 120, as calculated from plating homogenates of six injected flies, previously ground in TSB medium. Flies were kept at 30°C post-infection and transferred to fresh vials every 2 days. For scoring survival, the number of dead flies was recorded every 12 hours over a 4-day period. The experiment was repeated independently three times. Estimated survival curves were plotted from the raw data sets and the Log-rank (Mantel-Cox) test was used to determine significance between the curves. For clarity in display, 95% confidence intervals have been omitted from the graphs. The data was analyzed using GraphPad Prism 5 (GraphPad Software, Inc.); P<0.05 for two estimated survival curves was considered significant.

## Supporting Information

S1 Text Supporting Materials and Methods. Detailed description of materials and methods, and supporting Tables A-F.
(DOCX)

S1 Fig Schematic representation of strains constructed in this study. A schematic representation of the strains constructed during this study showing sequential deletion of genes encoding PG synthesis proteins in the intermediate and final mutant strains constructed to obtain COL MIN. The strain COL MIN lacks seven of the nine genes in the *S*. *aureus* genome that encode enzymes with TPase or TGase synthetic activity. Shaded white boxes represent transpeptidase activity, dark gray boxes indicate transglycosylase activity, light grey boxes suggest putative TPase activity and red crosses indicate deleted genes.
(TIF)

S2 Fig Confirmation of deletion of genes encoding PG synthesis enzymes in COL MIN. **(A)** PCR verification of gene deletions. Deletion of the genes encoding each of the PG synthesis enzymes was confirmed by PCR using primers flanking the deleted region. In COL (lanes a) the full-length gene was amplified and in COL MIN (lanes b) a shorter product was amplified for all genes, except those encoding PBP1 and PBP2, which were not deleted in COL MIN. **(B)** Western blot analysis of COL and COL MIN. Total protein extracts from COL and COL MIN were subjected to western blot analysis using PBP-specific antibodies. PBP1 and PBP2 proteins were present in both strains, while PBP3, PBP4 and PBP2A were undetectable in COL MIN.
(TIF)

S3 Fig *S*. *aureus* minimal mutant strain depleted for PBP1 can grow when PBP1 is expressed from a plasmid. Growth of the minimal mutant strain COL MIN PBP1ipSKP1 was followed in liquid medium by monitoring the absorbance at OD_600nm._ When depletion of PBP1 from COL MIN PBP1i is complemented by expression of PBP1 from the replicative plasmid pSKP1 cells grow normally in the presence or absence of IPTG (when PBP1 is no longer expressed from the P_*spac*_ promoter) showing that the insertion of the PBP1i construct into the genome does not cause lethal polar effects upon downstream genes.
(TIF)

S4 Fig Reduced secondary crosslinking of muropeptides observed in the minimal mutant strain COL MIN is due to lack of PBP4 and FmtA. HPLC profiles of muropeptides from *S*. *aureus* COL, COLΔ*pbpd*, COLΔ*fmtA* and COL MIN. Muropeptide elution profiles show that the secondary crosslinking notable in *S*. *aureus* is slightly reduced in the absence of FmtA and dramatically reduced in the absence of PBP4. The percentage of muropeptide species in the wild type, intermediate and COL MIN strains was quantified and is summarized in the table. To confirm the roles of PBP4 and FmtA in the reduction of secondary crosslinking, single deletion mutants of *pbpd* and *fmtA* were also analyzed. Experiments were repeated three times, chromatograms show results from one experiment. Values are displayed as a percentage of the total area of peaks analyzed.
(TIF)

S5 Fig Peptidoglycan composition of COL MIN shows normal glycan chain length. HPLC profile of glycan strands from *S*. *aureus* COL and COL MIN. Glycan strands were prepared from purified COL and COL MIN peptidoglycan digested with lysostaphin, a glycyl-glycine endopeptidase which digests the cross-bridges between muropeptides, followed by LytA amidase, which removes the peptides from the glycan strands. The profiles show glycans separated on the basis of size, shorter glycans eluting before longer glycans. Length and distribution of glycans in the COL MIN strain is unchanged in the absence of seven PG synthetic enzymes.
(TIF)

S6 Fig *S*. *aureus* minimal mutant strain Newman MIN displays normal growth. Growth of the parental MSSA strain Newman and the minimal mutant strain Newman MIN was followed in rich **(A)** and minimal **(B)** liquid medium by monitoring the absorbance at OD_600nm_. The mutant strain Newman MIN showed similar growth to the parental strain Newman in both cases.
(TIF)

S7 Fig Reduced growth of COL MIN mutant strain in healthy *Drosophila* flies. **(A)** The bacterial colony forming units (CFUs) per fly were determined at different time points of the infection. COL MIN was able to grow in an immunocompromised fly (PGRP-SA *seml*) but not in a wild-type fly background. Data shows mean with 95% confidence intervals. **(B) Quantification of** drosomycin expression at different time points of the infection, by RT-PCR. COL MIN induced drosomycin activation in WT flies although at levels lower than those induced by the parental strain COL. The results are expressed as means with 95% confidence intervals and are representative of three independent assays. Differences in the bacterial load and drosomycin expression between *S*. *aureus* strains over time were assessed by two-way ANOVA. Bonferroni post-tests were used to locate the time points where mean values were statistically separable between the two strains, significant differences are denoted by asterisk (** p<0.01; *** p<0.001).
(TIF)

## References

[1] Holtje JV (1996) A hypothetical holoenzyme involved in the replication of the murein sacculus of *Escherichia coli* . Microbiology 142: 1911–1918. 8760905 10.1099/13500872-142-8-1911

[2] Holtje JV (1998) Growth of the stress-bearing and shape-maintaining murein sacculus of *Escherichia coli* . Microbiology and Molecular Biology Reviews 62: 181–203. 9529891 10.1128/mmbr.62.1.181-203.1998 PMC98910

[3] Scheffers DJ, Pinho MG (2005) Bacterial cell wall synthesis: New insights from localization studies. Microbiology and Molecular Biology Reviews 69: 585–607. 16339737 10.1128/MMBR.69.4.585-607.2005 PMC1306805

[4] Hartman BJ, Tomasz A (1984) Low-Affinity Penicillin-Binding Protein Associated with Beta-Lactam Resistance in *Staphylococcus aureus* . Journal of Bacteriology 158: 513–516. 6563036 10.1128/jb.158.2.513-516.1984 PMC215458

[5] Pereira SFF, Henriques AO, Pinho MG, de Lencastre H, Tomasz A (2007) Role of PBP1 in cell division of *Staphylococcus aureus* . Journal of Bacteriology 189: 3525–3531. 17307860 10.1128/JB.00044-07 PMC1855886

[6] Pereira SFF, Henriques AO, Pinho MG, de Lencastre H, Tomasz A (2009) Evidence for a dual role of PBP1 in the cell division and cell separation of *Staphylococcus aureus* . Molecular Microbiology 72: 895–904. 10.1111/j.1365-2958.2009.06687.x 19400776 PMC2771448

[7] Pinho MG, Filipe SR, De Lencastre H, Tomasz A (2001) Complementation of the essential peptidoglycan transpeptidase function of penicillin-binding protein 2 (PBP2) by the drug resistance protein PBP2A in *Staphylococcus aureus* . Journal of Bacteriology 183: 6525–6531. 11673420 10.1128/JB.183.22.6525-6531.2001 PMC95481

[8] Pinho MG, de Lencastre H, Tomasz A (2001) An acquired and a native penicillin-binding protein cooperate in building the cell wall of drug-resistant staphylococci. Proceedings of the National Academy of Sciences of the United States of America 98: 10886–10891. 11517340 10.1073/pnas.191260798 PMC58569

[9] Pinho MG, de Lencastre H, Tomasz A (2000) Cloning, characterization, and inactivation of the gene *pbpC*, encoding penicillin-binding protein 3 of *Staphylococcus aureus* . Journal of Bacteriology 182: 1074–1079. 10648534 10.1128/jb.182.4.1074-1079.2000 PMC94384

[10] Curtis NAC, Hayes MV, Wyke AW, Ward JB (1980) A mutant of *Staphylococcus aureus* H lacking penicillin-binding protein 4 and transpeptidase activity *in vitro* . Fems Microbiology Letters 9: 263–266.

[11] Memmi G, Filipe SR, Pinho MG, Fu ZB, Cheung A (2008) *Staphylococcus aureus* PBP4 is essential for beta-lactam resistance in community-acquired methicillin-resistant strains. Antimicrobial Agents and Chemotherapy 52: 3955–3966. 10.1128/AAC.00049-08 18725435 PMC2573147

[12] Reed P, Veiga H, Jorge AM, Terrak M, Pinho MG (2011) Monofunctional transglycosylases are not essential for *Staphylococcus aureus* cell wall synthesis. Journal of Bacteriology 193: 2549–2556. 10.1128/JB.01474-10 21441517 PMC3133172

[13] Coutinho PM, Deleury E, Davies GJ, Henrissat B (2003) An evolving hierarchical family classification for glycosyltransferases. Journal of Molecular Biology 328: 307–317. 12691742 10.1016/s0022-2836(03)00307-3

[14] Kuroda M, Ohta T, Uchiyama I, Baba T, Yuzawa H, et al (2001) Whole genome sequencing of meticillin-resistant *Staphylococcus aureus* . Lancet 357: 1225–1240. 11418146 10.1016/s0140-6736(00)04403-2

[15] Komatsuzawa H, Ohta K, Sugai M, Fujiwara T, Glanzmann P, et al (2000) Tn551-mediated insertional inactivation of the f*mtB* gene encoding a cell wall-associated protein abolishes methicillin resistance in *Staphylococcus aureus* . Journal of Antimicrobial Chemotherapy 45: 421–431. 10896508 10.1093/jac/45.4.421

[16] Fan X, Liu YH, Smith D, Konermann L, Siu KWM, et al (2007) Diversity of penicillin-binding proteins—Resistance factor FmtA of *Staphylococcus aureus* . Journal of Biological Chemistry 282: 35143–35152. 17925392 10.1074/jbc.M706296200

[17] Komatsuzawa H, Ohta K, Labischinski H, Sugai M, Suginaka H (1999) Characterization of *fmtA*, a gene that modulates the expression of methicillin resistance in *Staphylococcus aureus* . Antimicrobial Agents and Chemotherapy 43: 2121–2125. 10471551 10.1128/aac.43.9.2121 PMC89433

[18] Boucher HW, Corey GR (2008) Epidemiology of methicillin-resistant *Staphylococcus aureus* . Clinical Infectious Diseases 46: S344–S349. 10.1086/533590 18462089

[19] Eddy SR (1998) Profile hidden Markov models. Bioinformatics 14: 755–763. 9918945 10.1093/bioinformatics/14.9.755

[20] Boneca IG, de Reuse H, Epinat JC, Pupin M, Labigne A, et al (2003) A revised annotation and comparative analysis of *Helicobacter pylori* genomes. Nucleic Acids Research 31: 1704–1714. 12626712 10.1093/nar/gkg250 PMC152854

[21] El Ghachi M, Mattei PJ, Ecobichon C, Martins A, Hoos S, et al (2011) Characterization of the elongasome core PBP2: MreC complex of *Helicobacter pylori* . Molecular Microbiology 82: 68–86. 10.1111/j.1365-2958.2011.07791.x 21801243

[22] Boneca IG, Ecobichon C, Chaput C, Mathieu A, Guadagnini S, et al (2008) Development of inducible systems to engineer conditional mutants of essential genes of *Helicobacter pylori* . Applied Environmental Microbiology 74: 2095–2102. 10.1128/AEM.01348-07 18245237 PMC2292604

[23] Matthews P, Tomasz A (1990) Insertional inactivation of the *mec* gene in a transposon mutant of a methicillin-resistant clinical isolate of *Staphylococcus aureus* . Antimicrobial Agents and Chemotherapy 34: 1777–1779. 2178337 10.1128/aac.34.9.1777 PMC171923

[24] Pereira AR, Reed P, Veiga H, Pinho MG (2013) The Holliday junction resolvase RecU is required for chromosome segregation and DNA damage repair in *Staphylococcus aureus* . BMC Microbiology 13: 18 10.1186/1471-2180-13-18 23356868 PMC3584850

[25] Tam R, Saier MH Jr. (1993) Structural, functional, and evolutionary relationships among extracellular solute-binding receptors of bacteria. Microbiology Reviews 57: 320–346. 8336670 10.1128/mr.57.2.320-346.1993 PMC372912

[26] Kuroda M, Kuroda H, Oshima T, Takeuchi F, Mori H, et al (2003) Two-component system VraSR positively modulates the regulation of cell-wall biosynthesis pathway in *Staphylococcus aureus* . Molecular Microbiology 49: 807–821. 12864861 10.1046/j.1365-2958.2003.03599.x

[27] Pinho MG, Errington J (2005) Recruitment of penicillin-binding protein PBP2 to the division site of *Staphylococcus aureus* is dependent on its transpeptidation substrates. Molecular Microbiology 55: 799–807. 15661005 10.1111/j.1365-2958.2004.04420.x

[28] Kuru E, Hughes HV, Brown PJ, Hall E, Tekkam S, et al (2012) *In situ* probing of newly synthesized peptidoglycan in live bacteria with fluorescent D-amino acids. Angewandte Chemie-International Edition 51: 12519–12523. 10.1002/anie.201206749 23055266 PMC3589519

[29] Pinho MG, Errington J (2003) Dispersed mode of *Staphylococcus aureus* cell wall synthesis in the absence of the division machinery. Molecular Microbiology 50: 871–881. 14617148 10.1046/j.1365-2958.2003.03719.x

[30] Steele VR, Bottomley AL, Garcia-Lara J, Kasturiarachchi J, Foster SJ (2011) Multiple essential roles for EzrA in cell division of *Staphylococcus aureus* . Molecular Microbiology 80: 542–555. 10.1111/j.1365-2958.2011.07591.x 21401734

[31] Wyke AW, Ward JB, Hayes MV, Curtis NAC (1981) A Role *in vivo* for Penicillin-Binding Protein 4 of *Staphylococcus aureus* . European Journal of Biochemistry 119: 389–393. 7308191 10.1111/j.1432-1033.1981.tb05620.x

[32] Boneca IG, Huang ZH, Gage DA, Tomasz A (2000) Characterization of *Staphylococcus aureus* cell wall glycan strands, evidence for a new beta-N-acetylglucosaminidase activity. Journal of Biological Chemistry 275: 9910–9918. 10744664 10.1074/jbc.275.14.9910

[33] Atilano ML, Yates J, Glittenberg M, Filipe SR, Ligoxygakis P (2011) Wall teichoic acids of *Staphylococcus aureus* limit recognition by the *Drosophila* peptidoglycan recognition protein-SA to promote pathogenicity. Plos Pathogens 7:E1002421 10.1371/journal.ppat.1002421 22144903 PMC3228820

[34] Kounatidis I, Ligoxygakis P (2012) *Drosophila* as a model system to unravel the layers of innate immunity to infection. Open Biology 2: 120075 10.1098/rsob.120075 22724070 PMC3376734

[35] Chang CI, Pili-Floury S, Herve M, Parquet C, Chelliah Y, et al (2004) A *Drosophila* pattern recognition receptor contains a peptidoglycan docking groove and unusual L,D-carboxypeptidase activity. PLoS Biology 2: E277 15361936 10.1371/journal.pbio.0020277 PMC515366

[36] Bera A, Biswas R, Herbert S, Kulauzovic E, Weidenmaier C, et al (2007) Influence of wall teichoic acid on lysozyme resistance in *Staphylococcus aureus* . Journal of Bacteriology 189: 280–283. 17085565 10.1128/JB.01221-06 PMC1797201

[37] Atilano ML, Pereira PM, Yates J, Reed P, Veiga H, et al (2010) Teichoic acids are temporal and spatial regulators of peptidoglycan cross-linking in *Staphylococcus aureus* . Proceedings of the National Academy of Sciences of the United States of America 107: 18991–18996. 10.1073/pnas.1004304107 20944066 PMC2973906

[38] Forsyth RA, Haselbeck RJ, Ohlsen KL, Yamamoto RT, Xu H, et al (2002) A genome-wide strategy for the identification of essential genes in *Staphylococcus aureus* . Molecular Microbiology 43: 1387–1400. 11952893 10.1046/j.1365-2958.2002.02832.x

[39] Labischinski H, Goodell EW, Goodell A, Hochberg ML (1991) Direct proof of a more-than-single-layered peptidoglycan architecture of *Escherichia coli* W7: a neutron small-angle scattering study. Journal of Bacteriology 173: 751–756. 1987162 10.1128/jb.173.2.751-756.1991 PMC207068

[40] Typas A, Banzhaf M, Gross CA, Vollmer W (2012) From the regulation of peptidoglycan synthesis to bacterial growth and morphology. Nature Reviews Microbiology 10: 123–136. 10.1038/nrmicro2677 22203377 PMC5433867

[41] Kelman Z, Odonnell M (1995) DNA-Polymerase-III Holoenzyme—Structure and function of a chromosomal replicating machine. Annual Review of Biochemistry 64: 171–200. 7574479 10.1146/annurev.bi.64.070195.001131

[42] Borukhov S, Nudler E (2003) RNA polymerase holoenzyme: structure, function and biological implications. Current Opinion in Microbiology 6: 93–100. 12732296 10.1016/s1369-5274(03)00036-5

[43] Giesbrecht P, Kersten T, Maidhof H, Wecke J (1998) Staphylococcal cell wall: Morphogenesis and fatal variations in the presence of penicillin. Microbiology and Molecular Biology Reviews 62: 1371–1414. 9841676 10.1128/mmbr.62.4.1371-1414.1998 PMC98950

[44] Goehring NW, Beckwith J (2005) Diverse paths to midcell: Assembly of the bacterial cell division machinery. Current Biology 15: R514–R526. 16005287 10.1016/j.cub.2005.06.038

[45] Vicente M, Rico AI (2006) The order of the ring: assembly of *Escherichia coli* cell division components. Molecular Microbiology 61: 5–8. 16824090 10.1111/j.1365-2958.2006.05233.x

[46] McPherson DC, Popham DL (2003) Peptidoglycan synthesis in the absence of class A penicillin-binding proteins in *Bacillus subtilis* . Journal of Bacteriology 185: 1423–1431. 12562814 10.1128/JB.185.4.1423-1431.2003 PMC142859

[47] Rice LB, Carias LL, Rudin S, Hutton R, Marshall S, et al (2009) Role of class A penicillin-binding proteins in the expression of beta-lactam resistance in *Enterococcus faecium* . Journal of Bacteriology 191: 3649–3656. 10.1128/JB.01834-08 19304851 PMC2681891

[48] Antignac A, Sieradzki K, Tomasz A (2007) Perturbation of cell wall synthesis suppresses autolysis in *Staphylococcus aureus*: evidence for coregulation of cell wall synthetic and hydrolytic enzymes. Journal of Bacteriology 189: 7573–7580. 17827298 10.1128/JB.01048-07 PMC2168716

[49] Strobel W, Moll A, Kiekebusch D, Klein KE, Thanbichler M (2014) Function and localization dynamics of bifunctional penicillin-binding proteins in *Caulobacter crescentus* . Journal of Bacteriology 196: 1627–1639. 10.1128/JB.01194-13 24532768 PMC3993357

[50] Sakoulas G, Okumura CY, Thienphrapa W, Olson J, Nonejuie P, et al (2014) Nafcillin enhances innate immune-mediated killing of methicillin-resistant *Staphylococcus aureus* . Journal of Molecular Medicine (Berlin) 92: 139–149. 10.1007/s00109-013-1100-7 24297496 PMC3926703

[51] Gough J, Karplus K, Hughey R, Chothia C (2001) Assignment of homology to genome sequences using a library of hidden Markov models that represent all proteins of known structure. Journal of Molecular Biology 313: 903–919. 11697912 10.1006/jmbi.2001.5080

[52] Wu M, Scott AJ (2012) Phylogenomic analysis of bacterial and archaeal sequences with AMPHORA2. Bioinformatics 28: 1033–1034. 10.1093/bioinformatics/bts079 22332237

[53] Guindon S, Gascuel O (2003) A simple, fast, and accurate algorithm to estimate large phylogenies by maximum likelihood. Systematic Biology 52: 696–704. 14530136 10.1080/10635150390235520

[54] Wu DY, Hugenholtz P, Mavromatis K, Pukall R, Dalin E, et al (2009) A phylogeny-driven genomic encyclopaedia of Bacteria and Archaea. Nature 462: 1056–1060. 10.1038/nature08656 20033048 PMC3073058

[55] Langmead B, Trapnell C, Pop M, Salzberg SL (2009) Ultrafast and memory-efficient alignment of short DNA sequences to the human genome. Genome Biology 10: R25 10.1186/gb-2009-10-3-r25 19261174 PMC2690996

[56] Filipe SR, Tomasz A, Ligoxygakis P (2005) Requirements of peptidoglycan structure that allow detection by the *Drosophila* Toll pathway. Embo Reports 6: 327–333. 15791270 10.1038/sj.embor.7400371 PMC1299281

[57] Jorge AM, Hoiczyk E, Gomes JP, Pinho MG (2011) EzrA contributes to the regulation of cell size in *Staphylococcus aureus* . Plos One 6:E27542 10.1371/journal.pone.0027542 22110668 PMC3215724

[58] Michel T, Reichhart JM, Hoffmann JA, Royet J (2001) *Drosophila* Toll is activated by Gram-positive bacteria through a circulating peptidoglycan recognition protein. Nature 414: 756–759. 11742401 10.1038/414756a

[59] Mackay TF, Richards S, Stone EA, Barbadilla A, Ayroles JF, et al (2012) The *Drosophila melanogaster* genetic reference panel. Nature 482: 173–178. 10.1038/nature10811 22318601 PMC3683990

