# Supplementary material for: Staphylococcus aureus Survives with a Minimal Peptidoglycan Synthesis Machine but Sacrifices Virulence and Antibiotic Resistance
Source: PLoS Pathog. 2015 May 7;11(5):e1004891. doi: 10.1371/journal.ppat.1004891 (PMC4423922; doi:10.1371/journal.ppat.1004891)
Supplement: S1 Text — Detailed description of materials and methods, and supporting Tables A-F. (DOCX) [file ppat.1004891.s001.docx]

**Supplementary Materials and Methods**

**Construction of *S. aureus strains***

Strains *S. aureus* COLΔ*pbp3*, COLΔ*pbpd*, and COLΔ*pbp3*Δ*pbpd* (COL Int 1) were already available [[1](#_ENREF_1)]. Deletion of *mgt* and *sgtA* from COL Int 1 was performed as previously described using plasmids pΔ*mgt* and pΔ*sgtA* [[2](#_ENREF_2)] and the resulting strain COLΔ*pbp3*Δ*pbpd*Δ*mgt*Δ*sgtA* was named COL Int 2. To construct an *fmtA* null mutant, we amplified 1kb DNA fragments from *S. aureus* COL genomic DNA [[3](#_ENREF_3)], corresponding to the upstream (primers FmtA-P1 and FmtA-P2) and downstream (primers FmtA-P3 and FmtA-P4) regions of the *fmtA* gene. The resulting PCR products were joined by overlap PCR using primers FmtA-P1 and FmtA-P4. The overlap PCR product was digested with NcoI and BamHI and cloned into the plasmid pMAD [[4](#_ENREF_4)], producing the plasmid pΔ*fmtA*. The plasmid was sequenced and introduced into RN4220 by electroporation [[5](#_ENREF_5)]. Following electroporation, the plasmid was transduced using phage 80α to COL and COL Int 2 strains using a previously described method [[6](#_ENREF_6)]. Insertion and excision of pΔ*fmtA* into the chromosome was performed as previously described [[4](#_ENREF_4)], resulting in strains COLΔ*fmtA,* and COLΔ*pbp3*Δ*pbpd*Δ*mgt*Δ*sgtA*Δ*fmtA* (COLΔ*34msf*).

For in-frame deletion of *fmtB*, 1kb from the upstream and downstream regions of the gene were amplified from COL genomic DNA by PCR using primer pairs pFmtB-KO-1/pFmtB-KO-2 and pFmtB-KO-3/pFmtB-KO-4, respectively. These fragments were joined by overlap PCR, digested with BamHI and EcoRI and cloned into the thermosensitive plasmid pMAD [[4](#_ENREF_4)] resulting in plasmid pΔ*fmtB*. The plasmid was introduced into strains COL and COLΔ*34msf*, integration and excision of the plasmid to delete *fmtB* was performed as described [[4](#_ENREF_4)], resulting in strains COLΔ*fmtB* and COLΔ*pbp3*Δ*pbpd*Δ*mgt*Δ*sgtA*Δ*fmtA*Δ*fmtB* (COL Int 3). Finally the *mecA* gene was deleted from strain COL Int 3, as previously described [[2](#_ENREF_2)], to construct the strain COLΔ*pbp3*Δ*pbpd*Δ*mgt*Δ*sgtA*Δ*fmtA*Δ*fmtB*Δ*mecA* (COL MIN). For immunofluorescence experiments the *spa* gene was deleted from COL and COL MIN using plasmid pMADspa [[7](#_ENREF_7)] using the same protocols described above, for the construction of strains COLΔ*spa* and COL MINΔ*spa*.

For construction of a *pbp1* conditional mutant, a fragment of the *pbp1* gene was amplified from NCTC8325-4 genomic DNA using primers PBP1inducible_BamP2 and PBP1induciblePspac_EcoP1. The PCR fragment was digested with BamHI and EcoRI then cloned into the same restriction sites in plasmid pMutin4 [[8](#_ENREF_8)]. Plasmid pMutin4PBP1t was introduced into RN4220 by electroporation and subsequently transduced to COL and COL MIN. The correct insertion of pMutin4PBP1t into the chromosome *via* a Campbell-type recombination event was confirmed by PCR, generating strains COLPBP1i and COL MIN PBP1i. Plasmid pSKP1 [[9](#_ENREF_9)], was transduced to COLPBP1i and COL MIN PBP1i as previously described [[9](#_ENREF_9)].

The construction of a *S. aureus* *pbp2* inducible mutant was already described [[10](#_ENREF_10)]. Introduction of the construct into COL and COL MIN was performed as previously described [[2](#_ENREF_2)]. Strains were verified by PCR and named COL PBP2i and COL MIN PBP2i respectively.

*S. aureus* Newman [[11](#_ENREF_11),[12](#_ENREF_12)] was used in order to make an MSSA minimal strain. The strain NewmanΔ*pbp3*Δ*pbpd* was already available [[1](#_ENREF_1)]. The genes encoding SgtA, MGT, FmtA and FmtB were deleted sequentially from this strain, as described above for COL. All gene deletions were confirmed by PCR and whole genome sequencing.

**Protein purification and antibody production**

The truncated *pbp1* gene lacking the sequence encoding the cytoplasmic N-terminal region and the putative transmembrane domain (M1-G44) was amplified from COL genomic DNA by PCR using primers PBP1p7B and PBP1p8x. The resulting PCR product was digested with BamHI and XhoI and cloned into the corresponding sites in the pET30a vector (Novagen), giving rise to pETPBP1t. This construct encodes an N-terminal His_6_ tag fusion of the truncated PBP1 (Q45-D744). *E. coli* BL21 (DE3) cells were transformed with pETPBP1t and the transformants were grown at 37^o^C in LB medium containing 50 μg/ml Kanamycin. When the absorbance at 600 nm reached 0.6 - 0.8, the cultures were supplemented with 1 mM IPTG and grown for an additional 3 hours. Cells were harvested by centrifugation, re-suspended in buffer A (50 mM sodium phosphate buffer pH 8 containing 150 mM NaCl and complete-EDTA-free protease inhibitors [Roche]) and were disrupted by sonication. After centrifugation at 48,000 xg at 4^o^C, the soluble fraction was applied to pre-equilibrated Talon^TM^ (Clontech) resin and incubated at 4^o^C overnight. Bound protein was washed twice with buffer A, and then once with buffer A containing 10 mM imidazole. Bound protein was eluted in a two-step manner with buffer A containing 100 mM then 150 mM imidazole. Eluted fractions were combined and dialyzed sequentially against 50 mM sodium phosphate buffer (pH 8) containing first 500 mM NaCl, then 300 mM NaCl, and finally 150 mM NaCl. The protein was 95% pure as visualized by SDS-PAGE and Coomassie staining. The purified protein was digested with thrombin to cleave the His_6_ tag, and separated on an 8% SDS-PAGE. The band corresponding to the truncated PBP1, minus the His_6_ tag, was cut from the gel and sent to Eurogentec (Belgium) for polyclonal antibody production.

Similar constructs for the expression and purification of PBP3, PBP4 and PBP2A were also made. For PBP3 cloning, primers PBP3-1 and PBP3-2 were used to amplify a truncated *pbp3* gene fragment missing the first 129 bp of the gene. The truncated gene encoded amino acids Q44-K648 and lacked the N-terminal cytoplasmic domain and the transmembrane region (L1-L43). A truncated *pbpd* gene lacking 60 5’, and 93 3’ base pairs was amplified from COL genomic DNA using the primer pair PBP4-1 and PBP4-2, and cloned into pET30a. A construct for the expression of a truncated version of PBP2A, lacking the N-terminal transmembrane domain (M1-Y23) was made using primers PBP2A-1 and PBP2A-2. The resulting constructs were sequenced and named pETPBP3t, pETPBP4t and pETPBP2At, respectively. *E. coli* BL21 (DE3) cells were transformed with the pET vectors, protein expression and purification were performed as described above for PBP1t. Bands containing protein without the His-tag were cut from SDS-PAGE gels as described above and sent for antibody production.

Polyclonal antibodies were tested for binding specificity against purified protein and whole cell extracts. The PBP3, PBP4 and PBP2A antibodies were further purified by incubation of the serum with the respective pure protein bound to PVDF membrane. Bound antibodies were released by incubation of the membrane with extraction buffer (0.2M HCL-Glycine, 0.2% BSA, pH 2.2) and the reaction stopped with 1M K_2_HPO_4_. The purified antibody solution was dialyzed against PBS containing 5% glycerol, and stored at 4^o^C.

**Western Blotting**

Absence of PBP3, PBP4 and PBP2A expression in the minimal mutant strain (COL MIN) was verified by western blotting. The parental strain COL and COL MIN were incubated at 37 ^o^C for 4 hours with aeration; cells were harvested and broken with glass beads in a FastPrep FP120 cell disrupter (Thermo Electro Corporation). Unbroken cells and debris were removed by centrifugation and total protein content of the clarified lysate was determined by the Bradford method using bovine serum albumin as a standard (BCA Protein Assay Kit, Pierce). Equal amounts of total protein from each sample were separated on 8% SDS-PAGE at 120V. Proteins were then transferred to Hybond-P PVDF membrane (GE Healthcare) using a BioRad Semi-dry transfer cell, according to standard western blotting techniques. PBP1, PBP2, PBP3, PBP4 and PBP2A proteins were detected using specific polyclonal antibodies.

**PCR verification of gene deletion**

In order to verify deletion of target genes from each mutant strain, genomic DNA was isolated and used for PCR amplification of the specific sequences. For each of the 9 (COL)/ 8 (Newman) genes involved in this study separate PCR reactions were set up using the following primers; PBP1Full-1 and PBP1Full-2 (*pbp*1); PBP2Full-1 and PBP2Full-2 (*pbp*2); pPBP3UpSeq and pPBP3DnRev (*pbp*3); pPBP4UpSeq and pPBP4DnRev (*pbpd*); pmecA-KO-P5 and pmecA-KO-P6 (mecA); MGT-P5 and MGT-P6 (mgt); pSgtA-P5 and pSgtA-p6 (*sgtA*); FmtA-11 and pFwdFmtAComp (*fmtA*); pFmtB-KO-5 and pFmtB-KO-6 (*fmtB*). Each of the primers hybridized in a region of the genome flanking the deleted gene, thus upon amplification either the full-length gene would be amplified or in the case of deletion mutant strains only a short fragment would result. The expected PCR product sizes were as follows; *pbp1* wild-type, 2241 bp; *pbp2* wild-type, 2184 bp; *pbp3* wild-type, 2686 bp, knock-out, 610 bp; *pbpd* wild-type, 1833 bp, knock-out, 537bp; *mecA* wild-type, 2299 bp, knock-out, 292 bp; *mgt* wild-type, 1136 bp, knock-out, 329 bp; *sgtA* wild-type, 1356 bp, knock-out, 410 bp; *fmtA* wild-type, 1500 bp, knock-out, 300bp; *fmtB* wild-type, 7882 bp, knock-out, 445bp.

**Structured illumination microscopy**

Overnight cultures of parental and mutant strains were diluted 1/500 and incubated at 37ºC. At mid-exponential phase (OD_600nm_ 0.5), 1 ml from each culture was taken and incubated with DNA dye Hoechst 33342 (1 µg/ml, Invitrogen) and the cell wall dye Van-FL (Invitrogen) mixed in a 1/1 (v/v) proportion with non-fluorescent vancomycin (4 µg/ml, Sigma) at room temperature for 5 minutes with shaking. Cells were harvested by centrifugation, re-suspended in PBS and 1 µl was placed on a thin layer of 1.2 % agarose in PBS. 2D-SIM images were obtained using a Zeiss Elyra PS.1 microscope with a SCMOS Pro SIM camera, using Zen software (Zeiss). Twenty-five images were taken (5 phases x 5 rotations) in two channels (405nm; 23 μm grating, 488nm; 28 μm grating) and reconstructions for each channel were performed using Zen software and theoretical PSF (point spread function). Quantifications and cell measurements were performed using image J.

**Immunofluorescence microscopy**

Strains COL Δ*spa* and COL MIN Δ*spa* were grown to an OD_600nm_ of 0.8 and culture samples (0.5 ml) were harvested and fixed with Histochoice (Amresco). Cells were washed three times with PBS then re-suspended in 500 μl of GTE buffer (50 mM glucose, 20 mM Tris-HCl, pH 7.5, 10 mM EDTA). A gentle lysis was performed using lysostaphin (Sigma) at a final concentration of 10 ng/ml for 1 min on a polylysine-treated slide. Cells were washed twice with PBS, air dried, rehydrated with PBS and blocked with 2% bovine serum albumin (BSA, Sigma) in PBS for 15 min. Cells were then incubated overnight at 4^o^C with primary antibody (anti-FtsZ, anti-PBP1 or anti-PBP2), which was added in consecutive two-fold dilutions from 1/800 to 1/3600. The following day cells were washed with PBS and incubated with secondary antibody (anti-Rabbit [PBP1 and PBP2] or anti-Sheep [FtsZ] diluted 1/500 in 2% BSA/PBS) in the dark for 1–2 hours. Cells were again washed with PBS, then Vectashield mounting medium (Vector Laboratories) was added and cells were visualized by fluorescence microscopy using an Axio Observer.Z1 microscope equipped with a Photometrics CoolSNAP HQ2 camera (Roper Scientific) and Metamorph software (Molecular Devices), and analyzed using Image J software.

**Electron microscopy**

S. aureus strains were grown in TSB until OD_600nm_ 0.7–0.8. Cells were harvested by centrifugation, washed with 0.1 M sodium-cacodylate buffer (pH 7.4) and fixed with 10 times their pellet volume of ice-cold 0.1 M sodium-cacodylate, 2.5% (v/v) glutaraldehyde buffer, pH 7.4. The cells were then centrifuged, washed with the same buffer, and post fixed with 2% osmium tetroxide for 2 h. After a brief rinse with buffer, the cells were dehydrated using a graded acetone series and embedded in Spurr medium [[13](#_ENREF_13)]. Thin sections were stained with uranyl acetate and lead citrate and viewed in a Philips BioTwin CM120 electron microscope at 100 kV. Individual micrographs were recorded on Kodak 4489 film at a nominal magnification of 11.000×.

**Peptidoglycan purification and analysis**

Peptidoglycan was prepared from exponentially growing cells as previously described [[14](#_ENREF_14)]. Muropeptides were prepared by digestion of PG with mutanolysin (Sigma), reduced with sodium borohydride (Sigma) and then analyzed by reverse-phase HPLC using a Hypersil ODS column (Thermo Electron Corporation). Glycan strands were isolated from purified peptidoglycan by sequential digestion with recombinant lysostaphin (1 μg/ml, Sigma) and purified pneumococcal amidase [[15](#_ENREF_15)] (LytA, 50-100 μg/ml) essentially as previously described [[16](#_ENREF_16)]. Glycan strands were separated from stem peptides on a MonoS column (GE Healthcare) in 10 mM sodium phosphate buffer pH 2, at 0.5 ml/min. Sample detection was followed at 202 nm and the glycan fraction was collected and concentrated approximately 10-fold to 500 μl. The samples were reduced with sodium borohydride and the pH adjusted to 2 with phosphoric acid. Reverse-phase HPLC was used to resolve the reduced glycans on a Nucleosil C18 column (Keystone Scientific) with a 0 - 10.5 % convex acetonitrile gradient in 100 mM sodium phosphate buffer pH 2. Glycan material was detected at 202 nm.

**Determination of colony forming units (CFUs) in *Drosophila*.** CFUs were determined at two different time points (0 and 24 hours) using six female flies. Flies were homogenized in TSB medium, serially diluted and plated onto TSB agar and incubated for 24 hours at 37°C.

**Drosomycin expression analysis in *Drosophila***. Total RNA was extracted from six female flies using the Total RNA Extraction Kit (Norgen) according to the manufacturer’s instructions. 500 ng of total RNA was used as a template for reverse cDNA transcription (SensiFast cDNA synthesis Kit, Bioline). Quantitative PCR reactions (SensiFast SYBR^R^ No-ROX Kit, Bioline) were carried out using 2 μl of ten-fold diluted cDNA template and 400 nM of each primer. Primer pair drs (+) GTACTTGTTCGCCCTCTTCG and drs (-) TTAGCATCCTTCGCACCAG was used to quantify drosomycin expression levels while primer pair tbp (+) GGCAAAGAGTGAGGACGACT and tbp (-) GAGCCGACCATGTTTTGAAT was used to measure the expression levels of the *tbp* gene. The housekeeping gene *tbp* [[17](#_ENREF_17)] was used as a control to normalize expression of the gene of interest. qPCR reactions were performed as outlined in the manufacturer’s instructions, amplicon amplification was performed with 40 cycles: 5 seconds at 95°C; 10 seconds at 62°C; 20 seconds at 72°C. Each reaction was performed in triplicate and three independent experiments were performed in a Quiagen Rotor-Gene Q real-time PCR cycler with a 72-well rotor. mRNA levels were calculated with the comparative CT method [[18](#_ENREF_18)].

**Supplementary References**

1. Memmi G, Filipe SR, Pinho MG, Fu ZB, Cheung A (2008) *Staphylococcus aureus* PBP4 is essential for beta-lactam resistance in community-acquired methicillin-resistant strains. Antimicrobial Agents and Chemotherapy **52**: 3955-3966.

2. Reed P, Veiga H, Jorge AM, Terrak M, Pinho MG (2011) Monofunctional transglycosylases are not essential for *Staphylococcus aureus* cell wall synthesis. Journal of Bacteriology **193**: 2549-2556.

3. Gill SR, Fouts DE, Archer GL, Mongodin EF, Deboy RT, *et al*. (2005) Insights on evolution of virulence and resistance from the complete genome analysis of an early methicillin-resistant *Staphylococcus aureus* strain and a biofilm-producing methicillin-resistant *Staphylococcus epidermidis* strain. Journal of Bacteriology **187**: 2426-2438.

4. Arnaud M, Chastanet A, Debarbouille M (2004) New vector for efficient allelic replacement in naturally nontransformable, low-GC-content, gram-positive bacteria. Applied and Environmental Microbiology **70**: 6887-6891.

5. Veiga H, Pinho MG (2009) Inactivation of the SauI type I restriction-modification system is not sufficient to generate *Staphylococcus aureus* strains capable of efficiently accepting foreign DNA. Applied and Environmental Microbiology **75**: 3034-3038.

6. Oshida T, Tomasz A (1992) Isolation and characterization of a Tn551-autolysis mutant of *Staphylococcus aureus*. Journal of Bacteriology **174**: 4952-4959.

7. Pereira PM, Veiga H, Jorge AM, Pinho MG (2010) Fluorescent reporters for studies of cellular localization of proteins in *Staphylococcus aureus*. Applied and Environmental Microbiology **76**: 4346-4353.

8. Vagner V, Dervyn E, Ehrlich SD (1998) A vector for systematic gene inactivation in *Bacillus subtilis*. Microbiology **144**: 3097-3104.

9. Pereira SFF, Henriques AO, Pinho MG, de Lencastre H, Tomasz A (2009) Evidence for a dual role of PBP1 in the cell division and cell separation of *Staphylococcus aureus*. Molecular Microbiology **72**: 895-904.

10. Pinho MG, Filipe SR, De Lencastre H, Tomasz A (2001) Complementation of the essential peptidoglycan transpeptidase function of penicillin-binding protein 2 (PBP2) by the drug resistance protein PBP2A in *Staphylococcus aureus*. Journal of Bacteriology **183**: 6525-6531.

11. Duthie ES, Lorenz LL (1952) Staphylococcal coagulase; mode of action and antigenicity. Journal of General Microbiolog **6**: 95-107.

12. Baba T, Bae T, Schneewind O, Takeuchi F, Hiramatsu K (2008) Genome sequence of *Staphylococcus aureus* strain Newman and comparative analysis of staphylococcal genomes: polymorphism and evolution of two major pathogenicity islands. Journal of Bacteriology **190**: 300-310.

13. Spurr AR (1969) A low-viscosity epoxy resin embedding medium for electron microscopy. Journal of Ultrastructure Research **26**: 31-43.

14. Filipe SR, Tomasz A, Ligoxygakis P (2005) Requirements of peptidoglycan structure that allow detection by the *Drosophila* Toll pathway. Embo Reports **6**: 327-333.

15. Garcia JL, Garcia E, Lopez R (1987) Overproduction and rapid purification of the amidase of *Streptococcus pneumoniae*. Archives of Microbiology **149**: 52-56.

16. Boneca IG, Huang ZH, Gage DA, Tomasz A (2000) Characterization of *Staphylococcus aureus* cell wall glycan strands, evidence for a new beta-N-acetylglucosaminidase activity. Journal of Biological Chemistry **275**: 9910-9918.

17. Matta BP, Bitner-Mathe BC, Alves-Ferreira M (2011) Getting real with real-time qPCR: a case study of reference gene selection for morphological variation in *Drosophila melanogaster* wings. Development Genes and Evolution **221**: 49-57.

18. Schmittgen TD, Livak KJ (2008) Analyzing real-time PCR data by the comparative C(T) method. Nature Protocols **3**: 1101-1108.

**Table A. Genomic mutations in COL MIN identified by whole genome sequencing.**

| **Genomic location** | **Description** | **Mutation** |
| --- | --- | --- |
| 39,633 | SACOL0033 (*mecA*) downstream | C-T |
| 39,635 | SACOL0033 (*mecA*) downstream | C-T |
| 41,660 | SACOL0033 (*mecA*) upstream | T (indel) |
| 309741  1075704  1175857  1436451  1436457  1638883  1820257  1820270  1996769  1996772  2222495  2334382 | SACOL0270 upstream  SACOL1066 (*fmtA*) downstream  SACOL1167 upstream  SACOL1425 upstream  SACOL1425 upstream  SACOL1608 upstream  SACOL1779 (*sgtA*) upstream  SACOL1779 (*sgtA*)  SACOL1932 (*mgt* last codon)  SACOL1932 (*mgt* last codon)  SACOL2150 (*fmtB* first codon)  SACOL2272 (*modA*) | G-T  C-T  C-T  T-A  G-A  A-G  T (indel)  A-T  A-G  A (indel)  A (indel)  T-A |

All mutations were identified by comparison to the parental strain COL.

**Table B. Differentially expressed transcripts in COL MIN (p<0.05 and over two-fold difference)**

| **Gene** | **Locus** | **Description** | **Fold change** | **p-value** | **Expression**  **up/down** |
| --- | --- | --- | --- | --- | --- |
| *purA* | SACOL0018 | purine nucleotide biosynthesis protein, PurA | 2.8 | 0.0195 | down |
| SACOL0199 | SACOL0199 | hypothetical membrane protein | 2.7 | 0.0479 | down |
| *uhpT* | SACOL0200 | sugar phosphate antiporter, UhpT | 3.7 | 0.01395 | down |
| *lrgA* | SACOL0247 | murein hydrolase regulator, LrgA | 4.4 | 0.002 | down |
| *lrgB* | SACOL0248 | antiholin-like protein, LrgB | 3.6 | 0.005 | down |
| *lytM* | SACOL0263 | peptidoglycan hydrolase, LytM | 3.3 | 0.00705 | down |
| SACOL0270 | SACOL0270 | staphyloxanthin biosynthesis protein, putative | 3 | 0.0275 | down |
| SACOL0323-0389 | SACOL0323-0389 | Prophage L54a | >2.5 | N/A | down |
| *ssb2* | SACOL0438 | single-stranded DNA-binding protein, SSB2 | 3.6 | 0.0081 | down |
| SACOL0501 | SACOL0501 | sodium-dependent transporter, putative | 2.9 | 0.01995 | down |
| SACOL0517 | SACOL0517 | alpha-amylase | 2.5 | 0.03495 | down |
| SACOL0772 | SACOL0772 | transcription regulator protein ExsB | 2.9 | 0.03715 | down |
| *dltA* | SACOL0935 | D-alanine--poly(phosphoribitol) ligase subunit 1, DltA | 2.7 | 0.034 | down |
| SACOL0946 | SACOL0946 | Na+/H+ antiporter family protein | 4.8 | 0.00075 | down |
| SACOL0947 | SACOL0947 | hypothetical protein | 3.9 | 0.00405 | down |
| *argH* | SACOL0963 | argininosuccinate lyase, ArgH | 2.7 | 0.0289 | down |
| *argG* | SACOL0964 | argininosuccinate synthase, ArgG | 2.8 | 0.0191 | down |
| *cydA* | SACOL1094 | cytochrome d ubiquinol oxidase, subunit I, CydA | 2.7 | 0.0248 | down |
| *cydB* | SACOL1095 | cytochrome d ubiquinol oxidase, subunit II, CydB | 2.6 | 0.03005 | down |
| SACOL1136 | SACOL1136 | hypothetical protein | 2.7 | 0.03455 | down |
| *trmD* | SACOL1256 | tRNA (guanine-N1)-methyltransferase, TrmD | 2.5 | 0.03875 | down |
| *glpF* | SACOL1319 | glycerol uptake facilitator protein, GlpF | 2.7 | 0.02055 | down |
| SACOL1344 | SACOL1344 | hypothetical protein | 550 | 0.00745 | down |
| **Gene** | **Locus** | **Description** | **Fold change** | **p-value** | **Expression**  **up/down** |
| SACOL1400 | SACOL1400 | ImpB/MucB/SamB family protein | 3.2 | 0.0207 | down |
| SACOL1481 | SACOL1481 | hypothetical protein | 3.2 | 0.0091 | down |
| SACOL1701 | SACOL1701 | hypothetical protein | 2.6 | 0.03335 | down |
| *thiI* | SACOL1764 | thiamine biosynthesis protein, ThiI | 2.4 | 0.04815 | down |
| SACOL1916 | SACOL1916 | amino acid ABC transporter permease/substrate-binding protein | 3 | 0.0216 | down |
| SACOL1983 | SACOL1983 | hypothetical protein | 26 | 0.047 | down |
| SACOL2088 | SACOL2088 | putative lytic transglycosylase, SceD | 3.6 | 0.00685 | down |
| SACOL2164 | SACOL2164 | hypothetical protein | 3.5 | 0.005 | down |
| SACOL2291 | SACOL2291 | staphyloxanthin biosynthesis protein, SsaA2 | 5.9 | 0.0006 | down |
| SACOL2295 | SACOL2295 | staphyloxanthin biosynthesis protein, putative | 4.5 | 0.00135 | down |
| SACOL2414 | SACOL2414 | amino acid ABC transporter, IraA | N/A | 0.014 | down |
| SACOL2581 | SACOL2581 | staphyloxanthin biosynthesis protein, polymorphism of SACOL2291 | 2.6 | 0.03 | down |
| *isaA* | SACOL2584 | putative lytic transglycosylase, IsaA | 2.8 | 0.0392 | down |
| *icaA* | SACOL2689 | N-glycosyltransferase, IcaA | 3.2 | 0.02405 | down |
| *hisD* | SACOL2702 | histidinol dehydrogenase, HisD | 3.8 | 0.0194 | down |
|  |  |  |  |  |  |
| *maoC* | SACOL0032 | MaoC domain protein | 8 | 5.00E-05 | up |
| *cap5A* | SACOL0136 | capsular polysaccharide biosynthesis protein Cap5A | 2.7 | 0.0327 | up |
| *cap5B* | SACOL0137 | capsular polysaccharide biosynthesis protein Cap5B | 2.6 | 0.03505 | up |
| *cap5C* | SACOL0138 | capsular polysaccharide biosynthesis protein Cap5C | 2.7 | 0.02895 | up |
| *cap5D* | SACOL0139 | capsular polysaccharide biosynthesis protein Cap5D | 2.6 | 0.0301 | up |
| *cap5I* | SACOL0144 | capsular polysaccharide biosynthesis protein Cap5I | 2.6 | 0.0383 | up |
| *cap5J* | SACOL0145 | capsular polysaccharide biosynthesis protein Cap5J | 2.6 | 0.04695 | up |
| *cap5K* | SACOL0146 | capsular polysaccharide biosynthesis protein Cap5K | 3 | 0.02375 | up |
| SACOL0156 | SACOL0156 | hypothetical protein | 3.84 | 0.0052 | up |
| **Gene** | **Locus** | **Description** | **Fold change** | **p-value** | **Expression**  **up/down** |
| SACOL0166 | SACOL0166 | hypothetical membrane protein, DUF1440 superfamily | 2.65 | 0.04035 | up |
| SACOL0191 | SACOL0191 | M23/M37 peptidase domain-containing protein | 2.6 | 0.04875 | up |
| *xpt* | SACOL0458 | xanthine phosphoribosyltransferase, Xpt | 4.5 | 0.0019 | up |
| *pbuX* | SACOL0459 | xanthine permease, PbuX | 4.4 | 0.00095 | up |
| SACOL0480 | SACOL0480 | hypothetical protein | 3.4 | 0.00725 | up |
| *vraX* | SACOL0625 | hypothetical protein, VraX | 11.6 | 5.00E-05 | up |
| SACOL0790 | SACOL0790 | integral membrane protein, transporter family protein | 30 | 0.0021 | up |
| SACOL0849 | SACOL0849 | hypothetical protein | 2.9 | 0.0307 | up |
| SACOL0851 | SACOL0851 | hypothetical protein | 2.7 | 0.02985 | up |
| SACOL0895 | SACOL0895 | pathogenicity island protein | 6 | 0.00915 | up |
| SACOL0948 | SACOL0948 | pyridine nucleotide-disulfide oxidoreductase | 13.2 | 5.00E-05 | up |
| SACOL1046 | SACOL1046 | hypothetical protein | 2.7 | 0.04715 | up |
| SACOL1071 | SACOL1071 | chitinase | 4.5 | 0.00135 | up |
| *purH* | SACOL1082 | bifunctional purine biosynthesis protein, PurH | 2.5 | 0.0438 | up |
| SACOL1087 | SACOL1087 | hypothetical protein | 3.3 | 0.0068 | up |
| SACOL1164 | SACOL1164 | fibrinogen binding-related protein | 2.7 | 0.02975 | up |
| SACOL1350 | SACOL1350 | hypothetical protein | 12.8 | 0.0181 | up |
| SACOL1705 | SACOL1705 | membrane protein | 10.6 | 5.00E-05 | up |
| SACOL1847 | SACOL1847 | hypothetical protein | 2.5 | 0.03995 | up |
| SACOL1853 | SACOL1853 | putative membrane protein | N/A | 0.037 | up |
| *epiG* | SACOL1871 | ABC-2 type transport system permease protein, EpiG | 2.9 | 0.039 | up |
| SACOL1896 | SACOL1896 | hypothetical protein | 3.5 | 0.0341 | up |
| *prsA* | SACOL1897 | protein export protein, PrsA | 3.6 | 0.00765 | up |
| *vraR* | SACOL1942 | DNA-binding response regulator, VraR | 3.2 | 0.01055 | up |
| *vraS* | SACOL1943 | sensor histidine kinase, VraS | 3.4 | 0.00645 | up |
| SACOL1944 | SACOL1944 | putative transporter, DUF2154 domain | 3.9 | 0.00385 | up |
| **Gene** | **Locus** | **Description** | **Fold change** | **p-value** | **Expression**  **up/down** |
|  |  |  |  |  |  |
| SACOL1945 | SACOL1945 | hypothetical protein | 5.9 | 0.00145 | up |
| SACOL1998 | SACOL1998 | membrane protein | 10 | 0.0317 | up |
| *hib* | SACOL2003 | phospholipase C, Hib | 3.6 | 0.00795 | up |
| SACOL2020 | SACOL2020 | nitroreductase | 2.9 | 0.02745 | up |
| SACOL2197 | SACOL2197 | surface protein | 2.9 | 0.02565 | up |
| *ureA* | SACOL2280 | urease subunit gamma, UreA | 4.4 | 0.01145 | up |
| *ureB* | SACOL2281 | urease subunit beta, UreB | 4.4 | 0.0052 | up |
| *ureC* | SACOL2282 | urease subunit alpha, UreC | 4.1 | 0.0021 | up |
| *ureE* | SACOL2283 | urease accessory protein UreE | 3.5 | 0.00885 | up |
| *ureF* | SACOL2284 | urease accessory protein, UreF | 3.8 | 0.00455 | up |
| *ureG* | SACOL2285 | urease accessory protein, UreG | 3.3 | 0.0103 | up |
| *ureD* | SACOL2286 | urease accessory protein, UreD | 3.7 | 0.00505 | up |
| SACOL2302 | SACOL2302 | transcriptional regulator | 2.6 | 0.0322 | up |
| SACOL2315 | SACOL2315 | hypothetical protein | 4.6 | 0.001 | up |
| SACOL2435 | SACOL2435 | glycerate kinase | 2.8 | 0.0171 | up |
| SACOL2436 | SACOL2436 | hypothetical membrane protein | 3.4 | 0.0062 | up |
| SACOL2450 | SACOL2450 | amino acid ABC transporter, permease protein | 2.8 | 0.0258 | up |
| SACOL2451 | SACOL2451 | amino acid ABC transporter, amino acid-binding protein | 2.7 | 0.0314 | up |
| SACOL2453 | SACOL2453 | amino acid ABC transporter, ATP-binding protein | 2.6 | 0.0408 | up |
| SACOL2484 | SACOL2484 | alkyl hydroperoxide reductase AhpD | 3.1 | 0.01215 | up |
| SACOL2538 | SACOL2538 | hypothetical protein | 3 | 0.0302 | up |
| SACOL2547 | SACOL2547 | membrane protein | 6 | 0.0041 | up |
| SACOL2571 | SACOL2571 | hypothetical protein, possibly exported | 26 | 5.00E-05 | up |
| *secY* | SACOL2675 | preprotein translocase subunit, SecY | 3.4 | 0.01815 | up |
| *sasA* | SACOL2676 | LPXTG cell wall surface anchor family protein, SasA | 2.9 | 0.0223 | up |

**Table C. MICs of parental, intermediate, and COL MIN strains to an array of antibiotics.**

| **Strain name** | **COL** | **COL**  **INT 1** | **COL**  **INT 2** | **COL**  **INT 3** | **COL MIN** |
| --- | --- | --- | --- | --- | --- |
| **Deleted enzymes** |  | 3,4 | 3,4,M,S | 3,4,M,S,Fa,Fb | 3,4,M,S,Fa,Fb,2A |
| **Antibiotic** |  |  |  |  |  |
| Oxacillin (TPases) | 800 | 800 | 400 | 3.125 | 3.125 |
| Imipenem (PBP1) | 50 | 50 | 50 | 0.39 | 0.39 |
| Cefotaxime (PBP2) | 500 | 500 | 250 | 3.9 | 3.9 |
| Cephradine (PBP3) | 500 | 250 | 62.5 | 3.9 | 3.9 |
| Cefoxitine (PBP4) | 500 | 500 | 250 | 3.9 | 3.9 |
| Flavomycin (TGases) | 2.5 | 0.31 | 0.31 | 0.31 | 0.31 |
| Bacitracin | 50 | 50 | 50 | 50 | 50 |
| Vancomycin | 2.5 | 2.5 | 2.5 | 2.5 | 2.5 |
| Lysostaphin | 0.125 | 0.125 | 0.125 | 0.125 | 0.125 |
| Fosfomycin | 1000 | 1000 | 1000 | 1000 | 1000 |
| Chloramphenicol | 5 | 5 | 5 | 5 | 5 |
| Naladixic Acid | 200 | 200 | 200 | 200 | 200 |

Key: Values represent MIC in μg/ml.

Deleted enzymes, PBP1 (1), PBP2 (2), PBP3 (3), PBP4 (4), MGT (M), SgtA (S), FmtA (Fa), FmtB (Fb) and PBP2A (2A).

**Table D. Strains used in this study**

| **Strain** | **Relevant Characteristics** | **Source/Reference** |
| --- | --- | --- |
| ***E. coli***  DH5α | F^-^, *end*A1, hsdR17 (r^-^, m^+^), *sup*E44, *thi*-1, λ-, *rec*A1, *gyr*A96, *rel*A1, φ80d*lac*ZΔM15 | Bethesda Research Laboratories |
| BL21 (DE3) | B F^-^ *dcm ompT hsdS (r_B_^-^m_B_^-^) gal* λ (DE3) | Stratagene |
| ***S. aureus*** |  |  |
| RN4220 | Restriction deficient derivative of NCTC8325-4 | R. Novick |
| Newman | MSSA clinical isolate | 11, 12 |
| COL | Homogeneous MRSA (MIC 1600 μg/ml) | 3 |
| COL PBP1i | COL with P_spac_-*pbp1* fusion in the chromosome, Ery^r^ | This study |
| RN4220 pPBP2iII | RN4220 with P_spac_-*pbp2* fusion in the chromosome transformed with pMGPII, Ery^r^ | 10 |
| COLIPBP2i | COL spa^-^ with P_spac_-*pbp2* fusion in the chromosome transformed with pMGPII, Tet^r^, Ery^r^ | 2 |
| COLΔ*pbp3* | COL *pbp3* null mutant | 1 |
| COLΔ*pbpd* | COL *pbpd* null mutant | 1 |
| COLΔ*mgt* | COL *mgt* null mutant | This study |
| COLΔ*sgtA* | COL *sgtA* null mutant | This study |
| COLΔ*fmtA* | COL *fmtA* null mutant | This study |
| COLΔ*fmtB* | COL *fmtB* null mutant | This study |
| COLΔ*mecA* | COL *mecA* null mutant | This study |
| COLΔ*spa* | COL *spa* null mutant | This study |
| COLΔ*mgt*Δ*sgtA* | COL *mgt* and *sgtA* null mutant | This study |
| COLΔ*pbp3*Δ*pbpd* (COL Int1) | COL *pbp3* and *pbpd* null mutant | 1 |
| COLΔ*pbp3*Δ*pbpd*Δ*mgt* | COL *pbp3,* *pbpd,* and *mgt* null mutant | This study |
| COLΔ*pbp3*Δ*pbpd*Δ*mgt*Δ*sgtA* (COLInt2) | COL *pbp3,* *pbpd, mgt,* and *sgtA* null mutant | This study |
| COLΔ*pbp3*Δ*pbpd*Δ*mgt*Δ*sgtA* Δ*fmtA* | COL *pbp3,* *pbpd, mgt, sgtA*, and *fmtA* null mutant | This study |
| COLΔ*pbp3*Δ*pbpd*Δ*mgt*Δ*sgtA* Δ*fmtA*Δ*fmtB* (COLInt3) | COL *pbp3,* *pbpd, mgt, sgtA*, *fmtA* and *fmtB* null mutant | This study |
| COLΔ*pbp3*Δ*pbpd*Δ*mgt*Δ*sgtA* Δ*fmtA*Δ*fmtB*Δ*mecA* (COL MIN) | COL *pbp3, pbpd, mgt, sgtA*, *fmtA,* *fmtB* and *mecA,* null mutant | This study |
| COL MIN Δ*spa* | COL MIN *spa* null mutant | This study |
| COL MIN PBP1i | COL MIN with P_spac_-*pbp1* fusion in the chromosome, Ery^r^ | This study |
| COL MIN PBP1i pSKP1 | COL MIN with P_spac_-*pbp1* fusion in the chromosome transformed with pSKP1, Ery^r^, Cm^r^ | This study |
| COL MIN PBP2i | COL MIN with P_spac_-*pbp2* fusion in the chromosome, Ery^r^ | This study |
| Newman Δ*pbp3*Δ*pbpd* | Newman *pbp3* and *pbpd* null mutant | 1 |
| Newman Δ*pbp3*Δ*pbpd*Δ*sgtA* | Newman *pbp3,* *pbpd,* and *sgtA* null mutant | This study |
| Newman Δ*pbp3*Δ*pbpd*Δ*sgtA* Δ*mgt* | Newman *pbp3,* *pbpd*, *sgtA* and *mgt* null mutant | This study |
| Newman Δ*pbp3*Δ*pbpd*Δ*sgtA* Δ*mgt*Δ*fmtA* | Newman *pbp3,* *pbpd*, *sgtA*, *mgt* and *fmtA* null mutant | This study |
| Newman Δ*pbp3*Δ*pbpd*Δ*sgtA* Δ*mgt*Δ*fmtA*Δ*fmtB* | Newman *pbp3,* *pbpd*, *sgtA*, *mgt*, *fmtA* and *fmtB* null mutant | This study |

**Table E: Plasmids used in this study**

| **Plasmid** | **Relevant Characteristics** | **Source/Reference** |
| --- | --- | --- |
| pMAD | *E. coli* (Ap^r^) – *S. aureus* (Ery^r^) shuttle vector | 3 |
| pMutin4 | S. aureus integrative vector, Amp^r^ Ery^r^ | 8 |
| pΔ*mgt* | pMAD with *mgt* up and downstream regions for constructing a null mutant, Ery^r^ | 2 |
| pΔ*sgtA* | pMAD with *sgtA* up and downstream regions for constructing a null mutant, Ery^r^ | 2 |
| pΔ*mecA* | pMAD with *mecA* up and downstream regions for constructing a null mutant, Ery^r^ | This study |
| p*Δspa* | pMAD with *spa* up and downstream regions for constructing a null mutant, Ery^r^ | 7 |
| pFmtAKO | pMAD with *fmtA* up and downstream regions for constructing a null mutant, Ery^r^ | This study |
| pFmtBKO | pMAD with *fmtB* up and downstream regions for constructing a null mutant, Ery^r^ | This study |
| pMutin4PBP1t | pMutin4 with truncated *pbp1* under control of the P*_spac_* promoter, Amp^r^ Ery^r^ | This study |
| pSKP1 | pSK5632 vector with wild-type *pbp1* copy, Ap^r^ Cm^r^ | 9 |
| pET30a | Cloning vector for producing His tag fusions, Kan^r^ | Novagen |
| pETPBP3t | pET30a expressing His-PBP3 | This study |
| pETPBP4t | pET30a expressing His-PBP4 | This study |
| pETPBP2At | pET30a expressing His-PBP2A | This study |
| pGL100 | Plasmid for the overexpression of pneumococcal amidase LytA, Amp^r^ | 15 |

**Table F: Primers used in this study.**

| **Primer** | **Sequence 5’-3’** | **Restriction site** |
| --- | --- | --- |
| pFmtA-1 | CTTACCATGGCGCGAAGTTGACAATTATCTTTGG | NcoI |
| pFmtA-2 | GCCATATACATGTTATATCTTCTATATCATTTGATAATTGCCTCAC |  |
| pFmtA-3 | GTGAGGCAATTATCAAATGATATAGAAGATATAACATGTATATGGC |  |
| pFmtA-4 | TATGGATCCctagagtgttcag | BamHI |
| pmecA_KO-P1 | tcggcacGAATTCtatacttaacacgttc | EcoRI |
| pmecA_KO-P2 | tcactgttttgcaatatcctccttatataagactac |  |
| pmecA_KO-P3 | taaggaggatattgcaaaacagtgaagcaatccgtaac |  |
| pmecA_KO-P4 | agcgcgtGGATCCgaatttaaatgtaga | BamHI |
| pmecA_KO-P5 | acacatatcgtgagcaatg |  |
| pmecA_KO-P6 | atggactcgttacagtgtc |  |
| pFmtB-KO-P1 | ACGCGAGAATTCCAATGAGAAATGTGGCTC | EcoRI |
| pFmtB-KO-P2 | ACTAGATGTTCACATTCTTAAGTCATCCTCCTG |  |
| pFmtB-KO-P3 | ACCAGGAGGATGACTTAAGAATGTGAACATCTAG |  |
| pFmtB-KO-P4 | AGCGCTGGATCCTCATTTTGCTAACTCAG | BamHI |
| pFmtB-KO-P5 | ACGCAGATGCTACTACTTATC |  |
| pFmtB-KO-P6 | AGTTGACGATGGCTTAGC |  |
| PBP1iPspac_EcoP1 | CTTGAATTCacgataatgtaaaggtag | EcoRI |
| PBP1iPspac_BamP2 | CTGGGGATCCgcatccatgacaaccgc | BamHI |
| PBP1p7B | CGCGGGATCCcaagatttagtcatgaaggc | BamHI |
| PBP1p8x | CCGCTCGAGTTAGTCCGACTTATCCTTGT | XhoI |
| pPBP3-1 | TATGGATCCCAAATCGCACAAGGCTCAC | BamHI |
| pPBP3-2 | GCCCGCCGAATTCTTATTTGTCTTTGTC | EcoRI |
| pPBP4-1 | GCGGATCCTATGCACAAGCTACTAACAG | BamHI |
| pPBP4-2 | CGCGAATTCTTATGGATGTTCTTCCCAC | EcoRI |
| pPBP2A-1 | CGCTCCGGATCCGCTTCAAAAGATAAAG | BamHI |
| pPBP2A-2 | GGCGAATTCCATCGTTACGGATTGCTTC | EcoRI |
| pPBP1Full-1 | tagcGGATCCgtagtaatggcgaagc | BamHI |
| pPBP1Full-2 | ttgGGATCCttagtccgacttatc | EcoRI |
| pPBP2Full-1 | tacGGATCCatgacggaaaacaaag | BamHI |
| pPBP2Full-2 | agcgcgcGAATCCttagttgaatatacc | EcoRI |
| pPBP3UPseq | acgccctgactacattg |  |
| pPBP3DNRev | tgtcatgatgaattatgcgac |  |
| pPBP4UPseq | tcgcttttgagtaagtttgctcttc |  |
| pPBP4DNRev | tgtcgatttagtcattccag |  |
| MGT-P5 | gtatgatggcgtatacgattg |  |
| MGT-P6 | gccacttcctatagcaaatg |  |
| SgtA-P5 | actagttcacagcgac |  |
| SgtA-P6 | acaacttcaagccaatc |  |
| pFwdFmtAComp | gttccttgtaatttgaatg |  |
| pFmtB-KO-5 | ACGCAGATGCTACTACTTATC |  |
| pFmtB-KO-6 | AGTTGACGATGGCTTAGC |  |
