# Supplementary figures and images for: Staphylococcus aureus Survives with a Minimal Peptidoglycan Synthesis Machine but Sacrifices Virulence and Antibiotic Resistance
Source: PLoS Pathog. 2015 May 7;11(5):e1004891. doi: 10.1371/journal.ppat.1004891 (PMC4423922; doi:10.1371/journal.ppat.1004891)

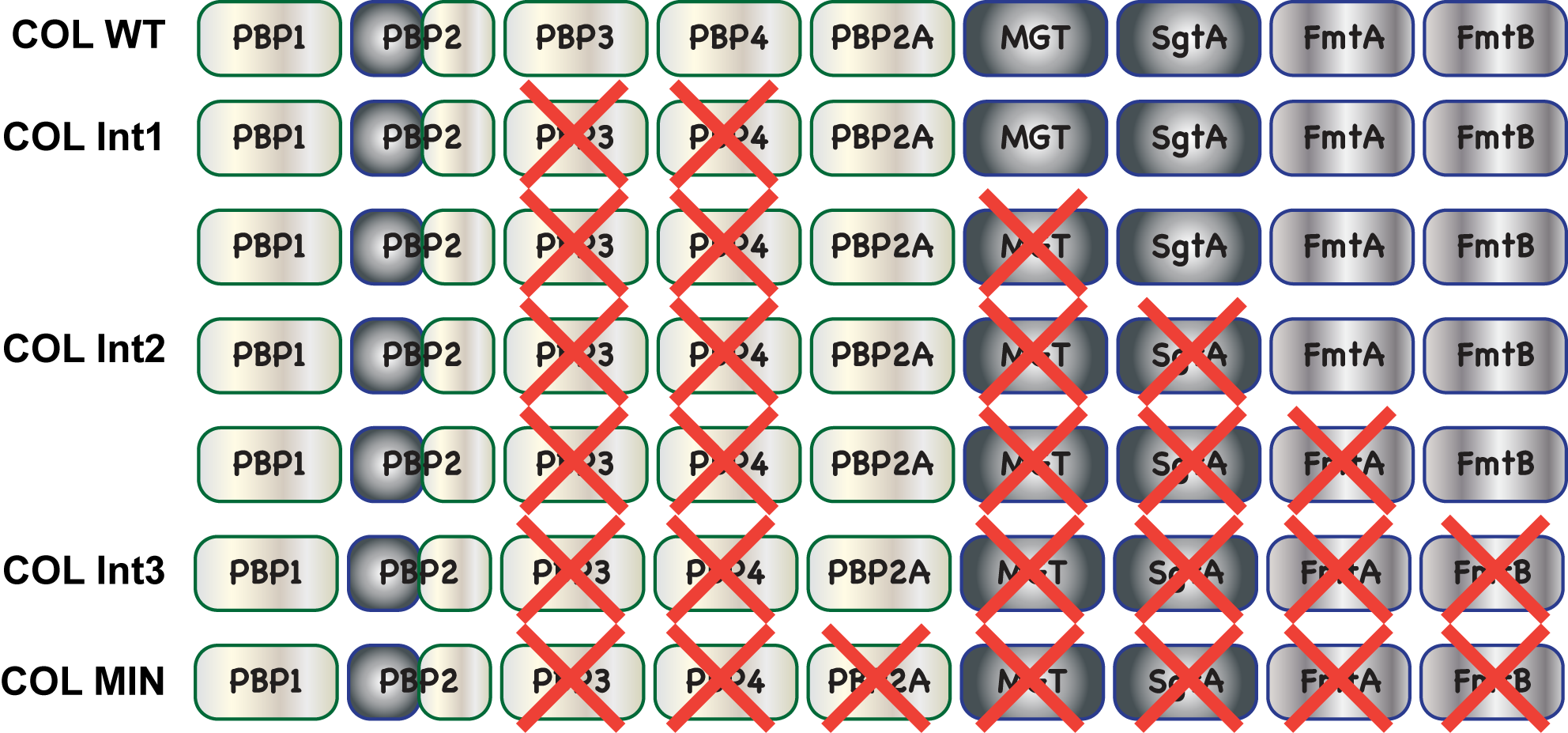

Supplement: S1 Fig — A schematic representation of the strains constructed during this study showing sequential deletion of genes encoding PG synthesis proteins in the intermediate and final mutant strains constructed to obtain COL MIN. The strain COL MIN lacks seven of the nine genes in the S. aureus genome that encode enzymes with TPase or TGase synthetic activity. Shaded white boxes represent transpeptidase activity, dark gray boxes indicate transglycosylase activity, light grey boxes suggest putative TPase activity and red crosses indicate deleted genes. (TIF) [file ppat.1004891.s002.tif]

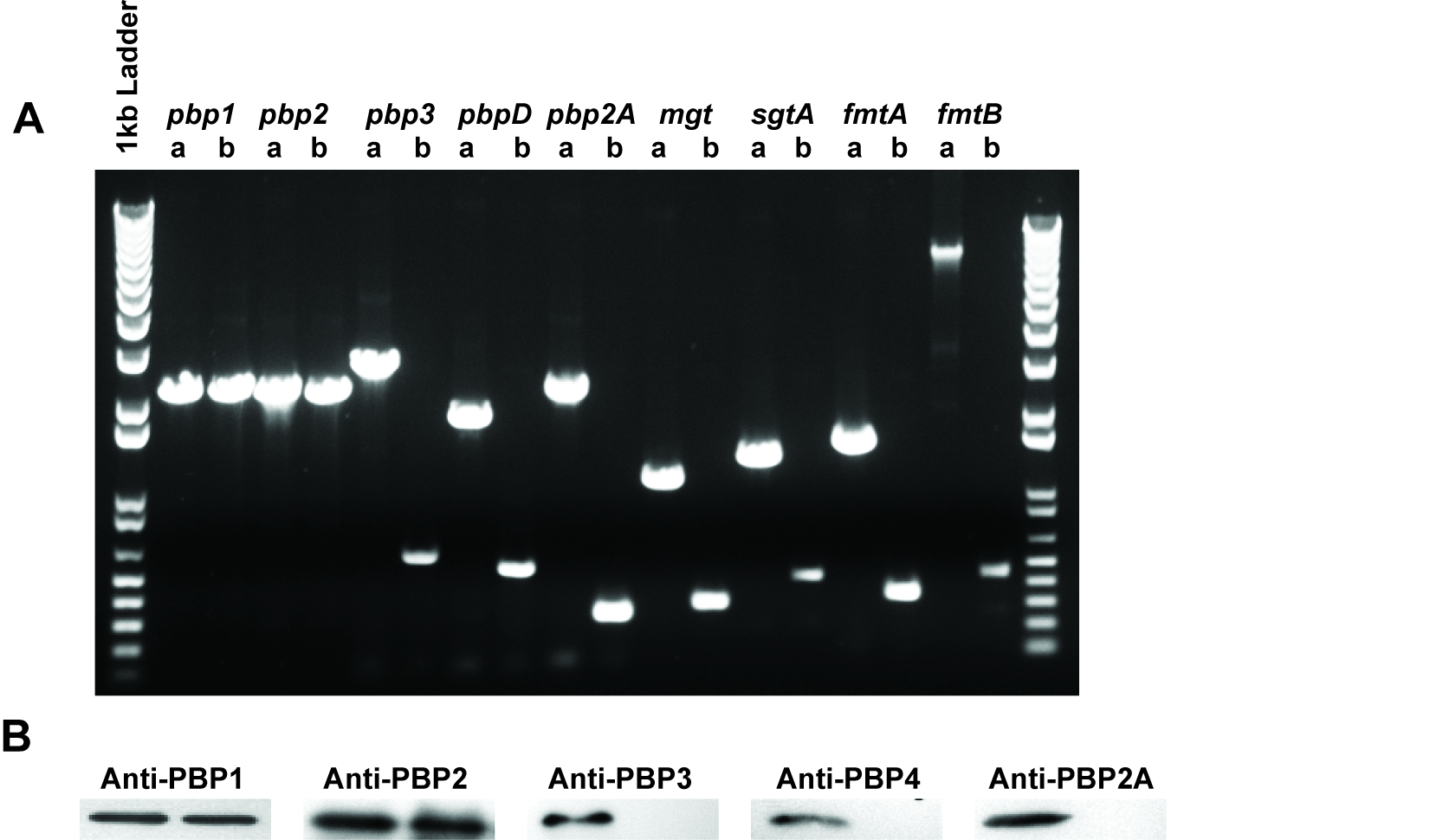

Supplement: S2 Fig — (A) PCR verification of gene deletions. Deletion of the genes encoding each of the PG synthesis enzymes was confirmed by PCR using primers flanking the deleted region. In COL (lanes a) the full-length gene was amplified and in COL MIN (lanes b) a shorter product was amplified for all genes, except those encoding PBP1 and PBP2, which were not deleted in COL MIN. (B) Western blot analysis of COL and COL MIN. Total protein extracts from COL and COL MIN were subjected to western blot analysis using PBP-specific antibodies. PBP1 and PBP2 proteins were present in both strains, while PBP3, PBP4 and PBP2A were undetectable in COL MIN. (TIF) [file ppat.1004891.s003.tif]

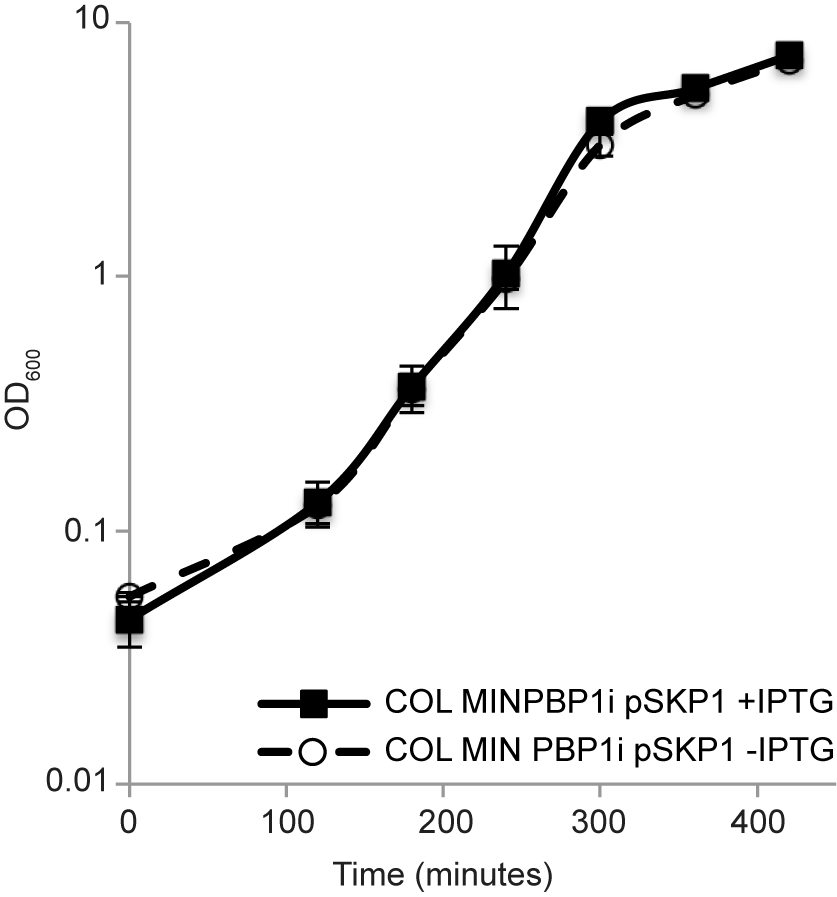

Supplement: S3 Fig — Growth of the minimal mutant strain COL MIN PBP1ipSKP1 was followed in liquid medium by monitoring the absorbance at OD600nm. When depletion of PBP1 from COL MIN PBP1i is complemented by expression of PBP1 from the replicative plasmid pSKP1 cells grow normally in the presence or absence of IPTG (when PBP1 is no longer expressed from the Pspac promoter) showing that the insertion of the PBP1i construct into the genome does not cause lethal polar effects upon downstream genes. (TIF) [file ppat.1004891.s004.tif]

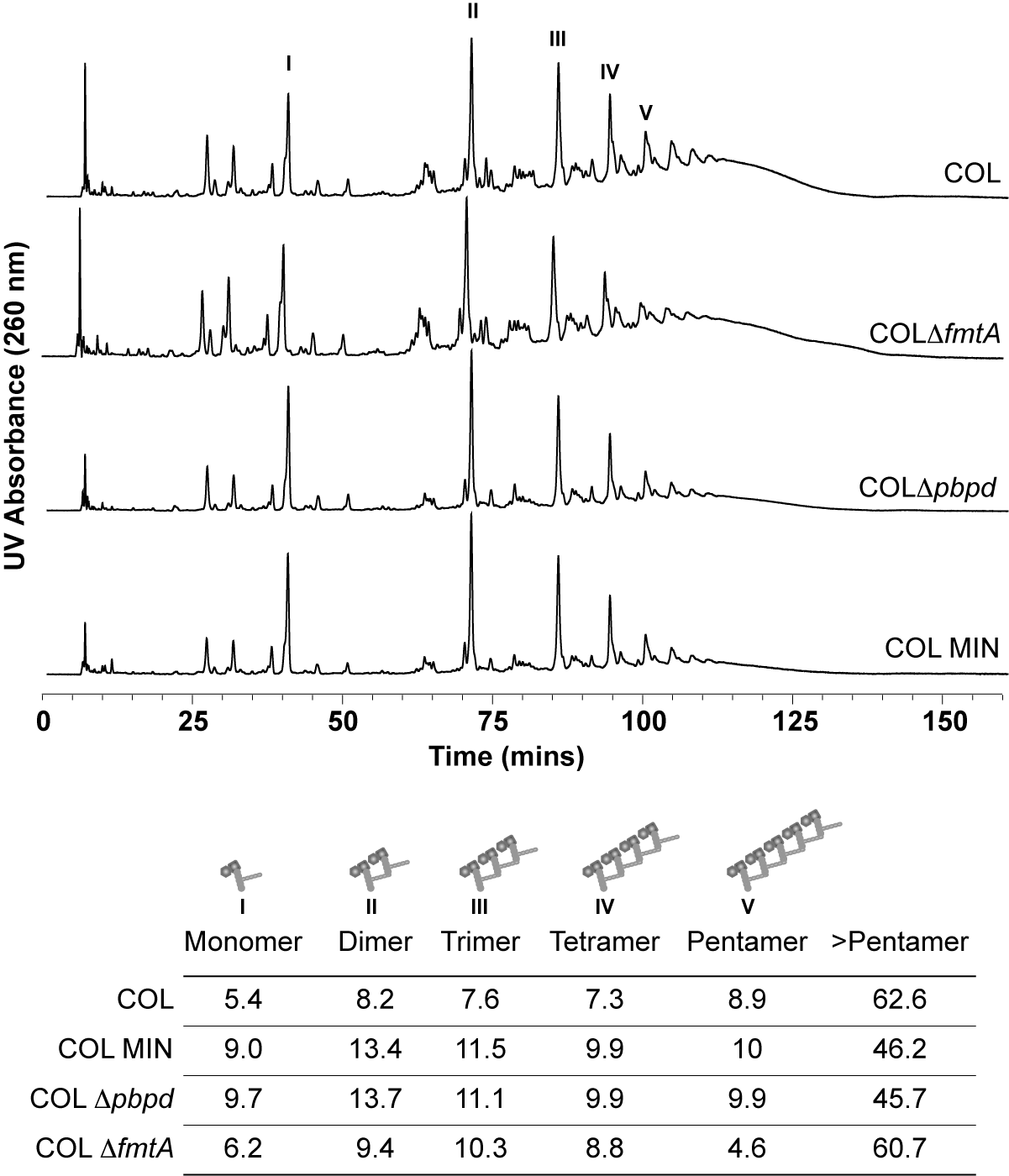

Supplement: S4 Fig — HPLC profiles of muropeptides from S. aureus COL, COLΔpbpd, COLΔfmtA and COL MIN. Muropeptide elution profiles show that the secondary crosslinking notable in S. aureus is slightly reduced in the absence of FmtA and dramatically reduced in the absence of PBP4. The percentage of muropeptide species in the wild type, intermediate and COL MIN strains was quantified and is summarized in the table. To confirm the roles of PBP4 and FmtA in the reduction of secondary crosslinking, single deletion mutants of pbpd and fmtA were also analyzed. Experiments were repeated three times, chromatograms show results from one experiment. Values are displayed as a percentage of the total area of peaks analyzed. (TIF) [file ppat.1004891.s005.tif]

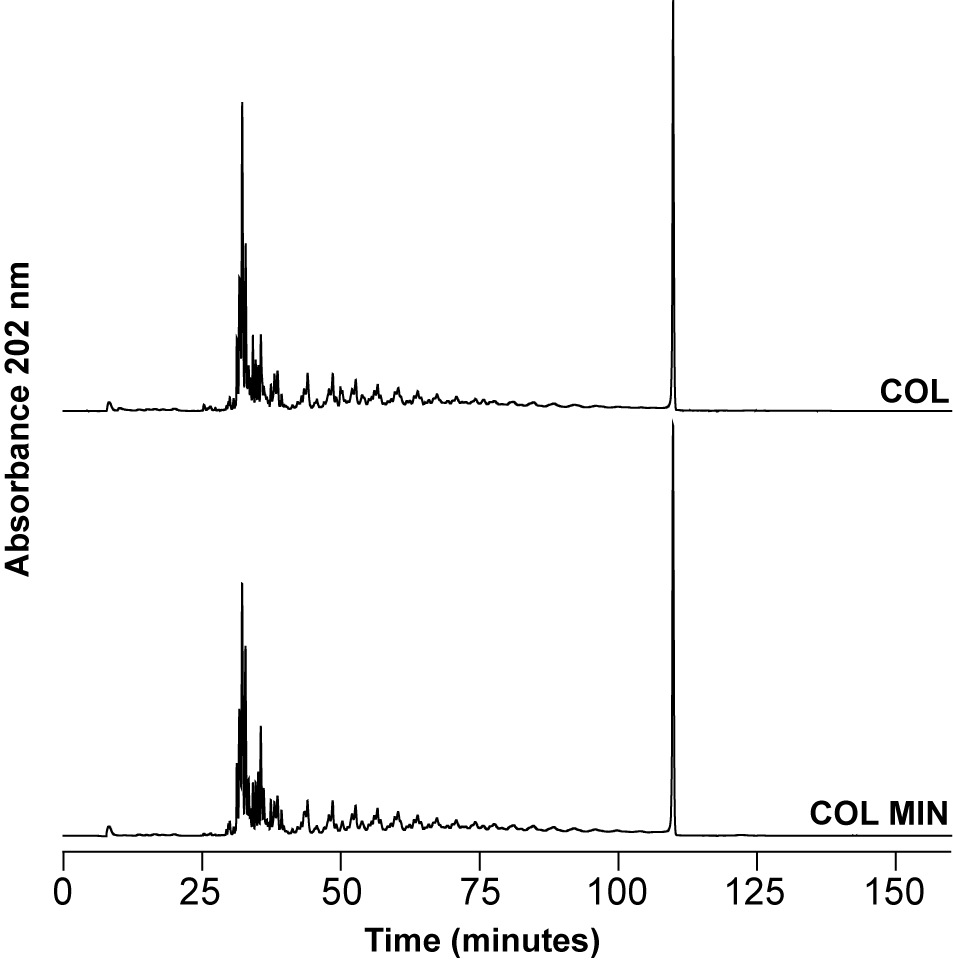

Supplement: S5 Fig — HPLC profile of glycan strands from S. aureus COL and COL MIN. Glycan strands were prepared from purified COL and COL MIN peptidoglycan digested with lysostaphin, a glycyl-glycine endopeptidase which digests the cross-bridges between muropeptides, followed by LytA amidase, which removes the peptides from the glycan strands. The profiles show glycans separated on the basis of size, shorter glycans eluting before longer glycans. Length and distribution of glycans in the COL MIN strain is unchanged in the absence of seven PG synthetic enzymes. (TIF) [file ppat.1004891.s006.tif]

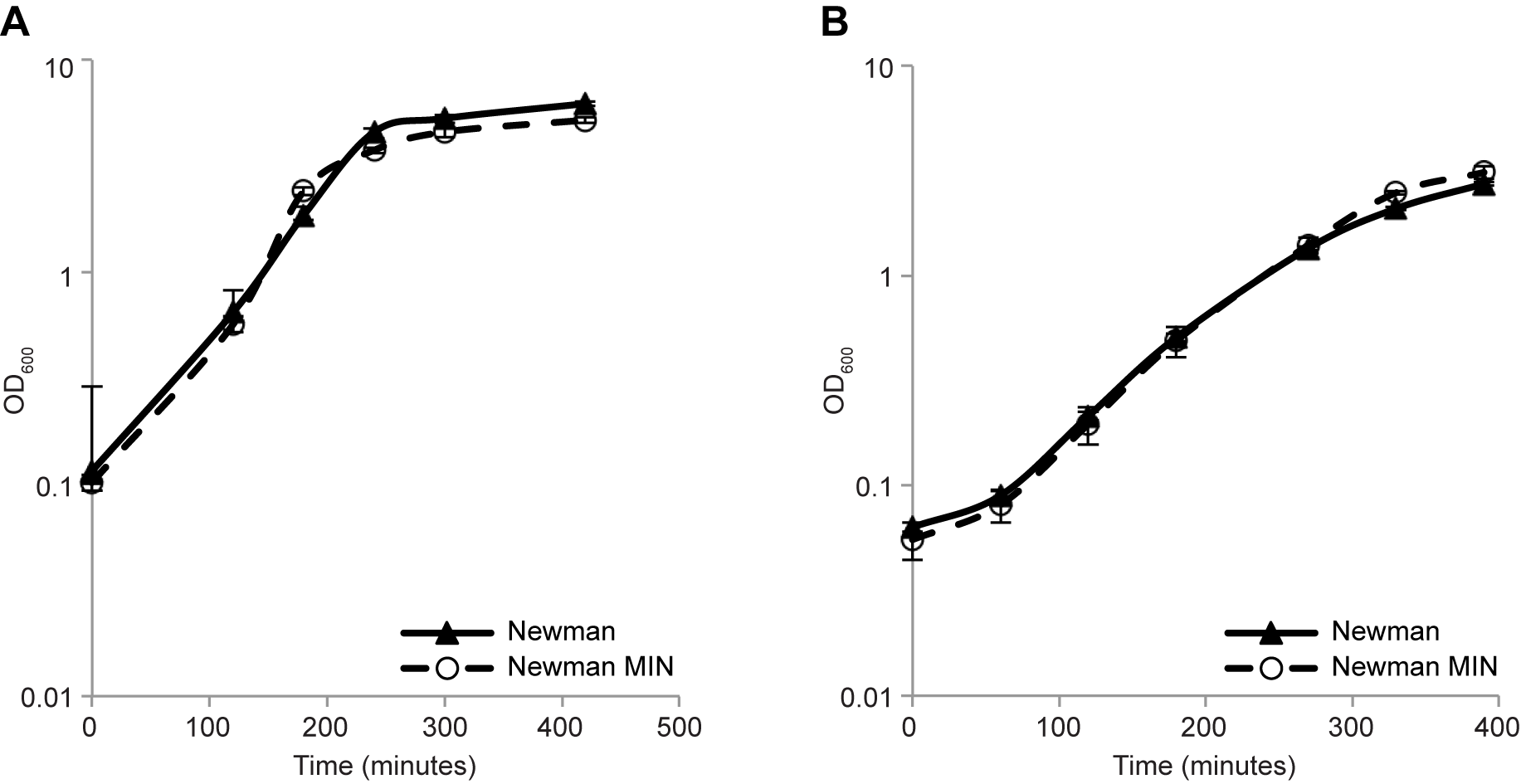

Supplement: S6 Fig — Growth of the parental MSSA strain Newman and the minimal mutant strain Newman MIN was followed in rich (A) and minimal (B) liquid medium by monitoring the absorbance at OD600nm. The mutant strain Newman MIN showed similar growth to the parental strain Newman in both cases. (TIF) [file ppat.1004891.s007.tif]

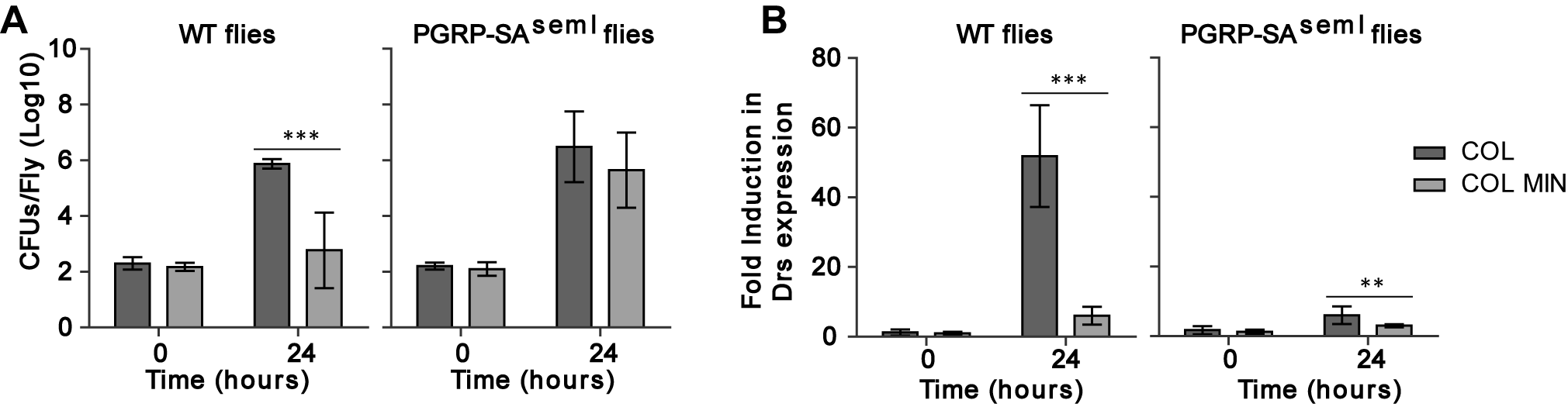

Supplement: S7 Fig — (A) The bacterial colony forming units (CFUs) per fly were determined at different time points of the infection. COL MIN was able to grow in an immunocompromised fly (PGRP-SA seml) but not in a wild-type fly background. Data shows mean with 95% confidence intervals. (B) Quantification of drosomycin expression at different time points of the infection, by RT-PCR. COL MIN induced drosomycin activation in WT flies although at levels lower than those induced by the parental strain COL. The results are expressed as means with 95% confidence intervals and are representative of three independent assays. Differences in the bacterial load and drosomycin expression between S. aureus strains over time were assessed by two-way ANOVA. Bonferroni post-tests were used to locate the time points where mean values were statistically separable between the two strains, significant differences are denoted by asterisk (** p<0.01; *** p<0.001). (TIF) [file ppat.1004891.s008.tif]
